# A Genetic Analysis of Tumor Progression in *Drosophila* Identifies the Cohesin Complex as a Suppressor of Individual and Collective Cell Invasion

**DOI:** 10.1016/j.isci.2020.101237

**Published:** 2020-06-04

**Authors:** Brenda Canales Coutiño, Zoe E. Cornhill, Africa Couto, Natalie A. Mack, Alexandra D. Rusu, Usha Nagarajan, Yuen Ngan Fan, Marina R. Hadjicharalambous, Marcos Castellanos Uribe, Amy Burrows, Anbarasu Lourdusamy, Ruman Rahman, Sean T. May, Marios Georgiou

**Affiliations:** 1 School of Life Sciences, University of Nottingham, Nottingham NG7 2UH, UK; 2 School of Biosciences, University of Nottingham, Sutton Bonington, Leicestershire LE12 5RD, UK; 3 Department of Biochemistry, School of Interdisciplinary and Applied Sciences, Central University of Haryana, Jant-Pali, Mahendergarh, Haryana, 123029, India; 4 Faculty of Biology, Medicine & Health, University of Manchester, Manchester M13 9PL, UK; 5 Department of Pharmacy and Pharmacology, University of Bath, Bath BA2 7AY, UK; 6 School of Medicine, University of Nottingham, Nottingham NG7 2UH, UK

**Keywords:** Biological Sciences, Molecular Biology, Cell Biology, Cancer

## Abstract

Metastasis is the leading cause of death for patients with cancer. Consequently it is imperative that we improve our understanding of the molecular mechanisms that underlie progression of tumor growth toward malignancy. Advances in genome characterization technologies have been very successful in identifying commonly mutated or misregulated genes in a variety of human cancers. However, the difficulty in evaluating whether these candidates drive tumor progression remains a major challenge. Using the genetic amenability of *Drosophila melanogaster* we generated tumors with specific genotypes in the living animal and carried out a detailed systematic loss-of-function analysis to identify conserved genes that enhance or suppress epithelial tumor progression. This enabled the discovery of functional cooperative regulators of invasion and the establishment of a network of conserved invasion suppressors. This includes constituents of the cohesin complex, whose loss of function either promotes individual or collective cell invasion, depending on the severity of effect on cohesin complex function.

## Highlights

• Screen identifies genes that affect tumor behavior in a wide variety of ways
• A functionally validated network of invasion-suppressor genes was generated
• Loss of cohesin complex function can promote individual or collective cell invasion
• The fly pupal notum is an excellent *in vivo* system to study tumor progression

## Graphical Abstract

**Figure:**
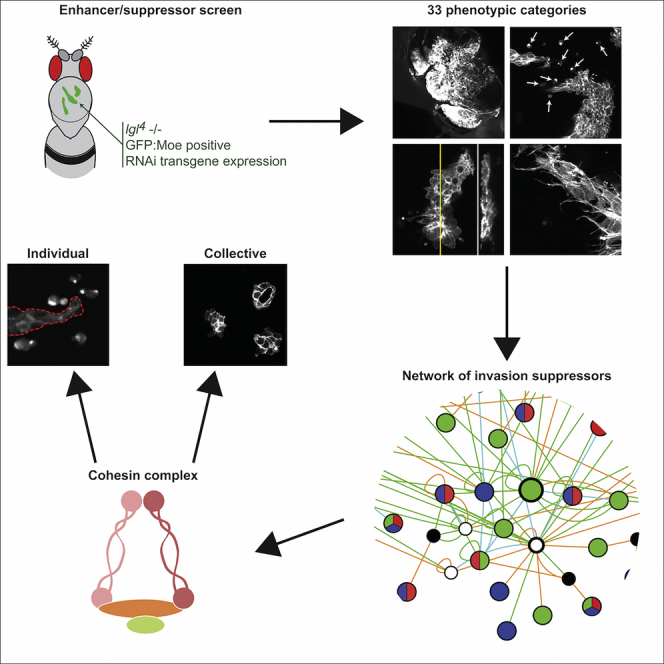

## Introduction

Metastasis is the major cause of mortality in human cancers, yet we know relatively little about the biology that underlies the important transition to invasive malignancy (Sporn, 1996, Hanahan and Weinberg, 2000) and currently few genes have been identified that suppress this process (Steeg, 2003, Yan et al., 2013). Most human cancers are epithelial in origin; consequently cancer cell invasion, where individual cells or groups of cells break away from the primary tumor to invade the surrounding tissue, is a key hallmark of tumor progression. Invasion is highly complex, involving concurrent dramatic changes in cytoskeletal organization, cell polarity, cell-cell junctions, and focal contacts, as cells within the developing tumor collectively destroy the normal architecture of the host epithelium and deregulate the local microenvironment (Hurst and Welch, 2011). Understanding and dissecting the molecular mechanisms that promote tumor progression and cancer cell invasion will be important for the development of new therapeutic strategies in our battle against this disease.

*Drosophila melanogaster* has become an increasingly important model system in the study of cancer biology. Conservation of major signaling pathways related to tumorigenesis and metastasis, coupled with the genetic amenability of this organism, has directly led to advances in our understanding of this disease (Rudrapatna et al., 2012, Brumby and Richardson, 2005). The short lifespan and low running costs of this organism make it particularly amenable to large-scale screens, and there is now a vast array of published literature using the fly to study cancer (Gonzalez, 2013, Rudrapatna et al., 2012, Mirzoyan et al., 2019).

We have developed an *in vivo* system in *Drosophila* that allows us to study epithelial cell and tissue morphogenesis in real time (Georgiou et al., 2008, Georgiou and Baum, 2010, Cohen et al., 2010, Couto et al., 2017). This system allows the shape, dynamics, and behavior of labeled mutant epithelial cells to be followed in high resolution in the living animal. In this current study, we use this *in vivo* system to generate tumors with specific genotypes on the dorsal thorax epithelium of the fly and to observe tumor cell morphology and behavior in high spatial and temporal resolution. Although several large-scale cancer screens have been carried out in the fly (for example, Moberg et al., 2001, Tapon et al., 2001, Woodhouse et al., 2003, Pagliarini and Xu, 2003, Zoranovic et al., 2018), our focus was to image and detail primary tumor behavior and progression in the living animal. By combining sophisticated *Drosophila* genetic techniques with transgenic RNAi technology we present here a detailed systematic loss-of-function (LOF) analysis that has identified genes that enhance or suppress tumor progression in this epithelium. We identify a number of conserved invasion suppressors that promote tumor cell invasion upon loss of expression. We further characterize components of the cohesin complex, which we find to be an important invasion suppressor and show that cohesin LOF can promote either individual or collective cell invasion, depending on the subunit that is mutated and the degree of effect on cohesin function.

## Results

We developed an *in vivo* genetic system in the fly that allows us to (1) generate a patch of tissue on the dorsal thorax that is homozygous mutant for a tumor suppressor, surrounded by wild-type (WT) tissue; (2) specifically label the mutant tissue with GFP:Moe (the actin-binding domain of moesin fused to GFP), thereby labeling the actin cytoskeleton of these cells; and (3) overexpress an RNAi transgene to deplete expression of a gene of interest specifically within the mutant, labeled tissue. Coupled with our ability to image this epithelium in the living animal in high temporal and spatial resolution (Couto et al., 2017), this system allowed us to conduct a large-scale genetic screen to identify genes that affect tumor behavior and tumor progression in a wide variety of ways.

### Design of an *In Vivo* Assay to Identify Modulators of Epithelial Tumor Progression

**Figure 1:**
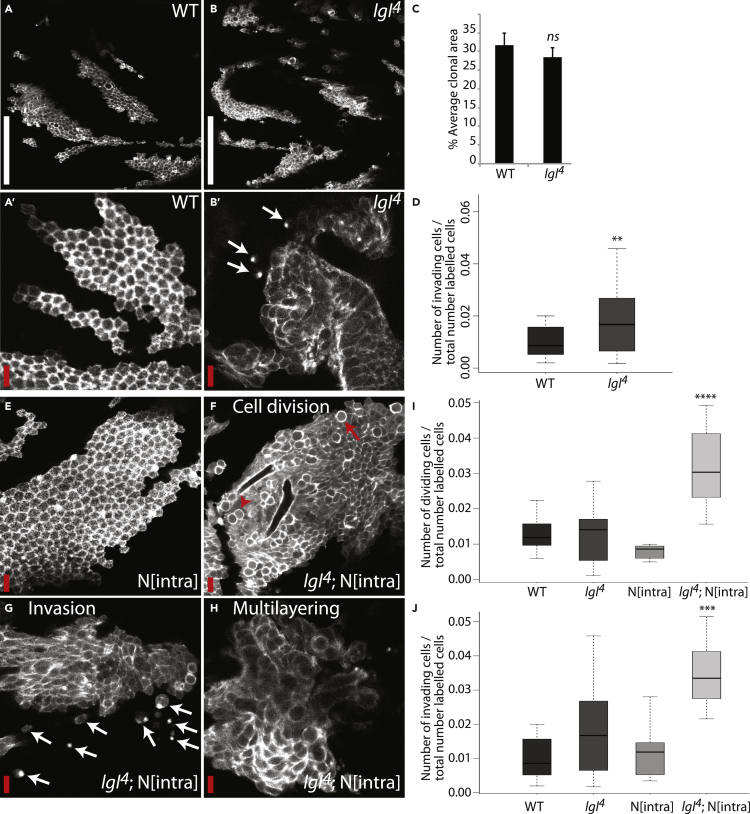
*lgl*^*4*^ Mutant Clones Provide an Ideal Genetic Background for an Enhancer/Suppressor Screen for Tumor Progression (A and B) GFP:Moe-labeled genetic clones in the dorsal thorax epithelium of living fly pupae. Clones shown are wild-type (A and A′) or homozygous mutant for the neoplastic tumor suppressor *lgl* (B and B′). (C and D) Quantification of average clonal area (C) (n = 10 [WT]; 18 [*lgl*^*4*^]) and the number of invading cells/the total number of labeled cells (D) (n = 30 [WT]; 41 [*lgl*^*4*^]). Quantification shows *lgl*^*4*^ mutant clones to be similar to WT clones in size, with a significant increase in the number of invading cells. (E–H) GFP:Moe-labeled genetic clones in the dorsal thorax epithelium of living fly pupae. Clones shown are overexpressing activated Notch (N^intra^; (E) or simultaneously homozygous mutant for *lgl*^*4*^ and overexpressing N^intra^ (F–H). Highlighted are effects on cell division (F), invasion (G), and multilayering (H). (I and J) Quantification of the number of dividing cells (I) and the number of invading cells (J) over the total number of labeled cells for clones with the genotypes shown (n = 30 [WT]; 41 [*lgl*^*4*^]; 7 [N^intra^]; 13 [*lgl*^*4*^; N^intra^]). Error bars represent ± SEM. Student's t test (E) and Kruskall-Wallis test (F, K, and L) were performed to determine statistical significance. p > 0.05 was considered not significant, ∗p < 0.05, ∗∗p < 0.01, ∗∗∗p < 0.001, ∗∗∗∗p < 0.0001. Red arrow, dividing cell; red arrowhead, cell doublet following cytokinesis; white arrows, invading cells. White scale bar, 50 μm; red scale bar, 10 μm.

We combined the Flp/FRT system (Xu and Rubin, 1993) the MARCM technique (Lee and Luo, 2001), and Pannier-Gal4 to generate positively marked homozygous mutant clones specifically within the epithelium of the fly pupal notum (the dorsal thorax). When imaging GFP:Moe-labeled WT clones within the pupal notum (at 20–24 h APF [after puparium formation]) we observed columnar epithelial cells that formed an organized monolayer on the back of the fly (Figure 1A and A′). Preparatory experiments identified *lethal (2) giant larvae*^*4*^ homozygous mutant clones (*lgl*^*4*^) as a suitable genetic background for our screen, as tumors lacking lgl were large, partially multilayered, and presented a low-level invasive phenotype, representing an ideal scenario for an enhancer/suppressor screen (Figures 1B–1D). Lgl is highly conserved, critical for the correct maintenance of cell polarity, and has also been found to control tissue growth and differentiation (Stephens et al., 2018). Lgl is a member of the scribble polarity complex, constituents of which (lgl, scribble, dlg) have been termed “neoplastic” tumor suppressors because mutations in these genes can generate highly disorganized multilayered tumors that are immortal, fail to differentiate, and show a high metastatic potential upon transplantation (Bilder, 2004, Gateff, 1978). In addition, expression of scribble complex genes has been shown to be lost or downregulated in numerous types of human cancer (Lo et al., 2012).

Although multilayered, amorphous, and invasive overgrowth is observed in *lgl*, *scribble,* or *dlg* mutant tissue, overgrowth is not observed when small mutant clones are generated, surrounded by WT tissue; here clones are restrained from overgrowth via a process known as “cell competition.” Mutant cells, despite undergoing excessive cell proliferation, are eliminated from the epithelium by Jun N-terminal kinase (JNK) pathway-mediated apoptosis (Brumby and Richardson, 2003, Nagata and Igaki, 2018). Both *scribble* and *lgl*^*4*^ mutants have previously been shown to cooperate with oncogenic Notch overexpression to overcome the effects of cell competition and cause neoplastic overgrowths within the proliferative epithelial primordia known as the *imaginal discs* (Brumby and Richardson, 2003, Khan et al., 2013). We wanted to see whether we could observe a similar cooperative effect within the pupal notum, which at the developmental stage of our analysis (20–24 h APF), is largely post-mitotic. When generating GFP:Moe-labeled clones of cells expressing activated Notch (N^intra^) in the notum, we observed relatively normal clones, with no effect either on cell shape or on tissue organization, and with no invasive characteristics (Figures 1E and 1I–J). When overexpressing N^intra^ specifically within *lgl*^*4*^ clones, however, we observed a strong cooperative effect—these clones showed strong hyperproliferation, with increased levels of cell division, loss of normal epithelial architecture, and increased invasion when compared with *lgl*^*4*^ alone (Figures 1F–1J). We therefore had generated an *in vivo* system that would allow us to identify mutations that work cooperatively with *lgl*^*4*^ to promote tumor progression.

### Pilot Screen

**Figure 2:**
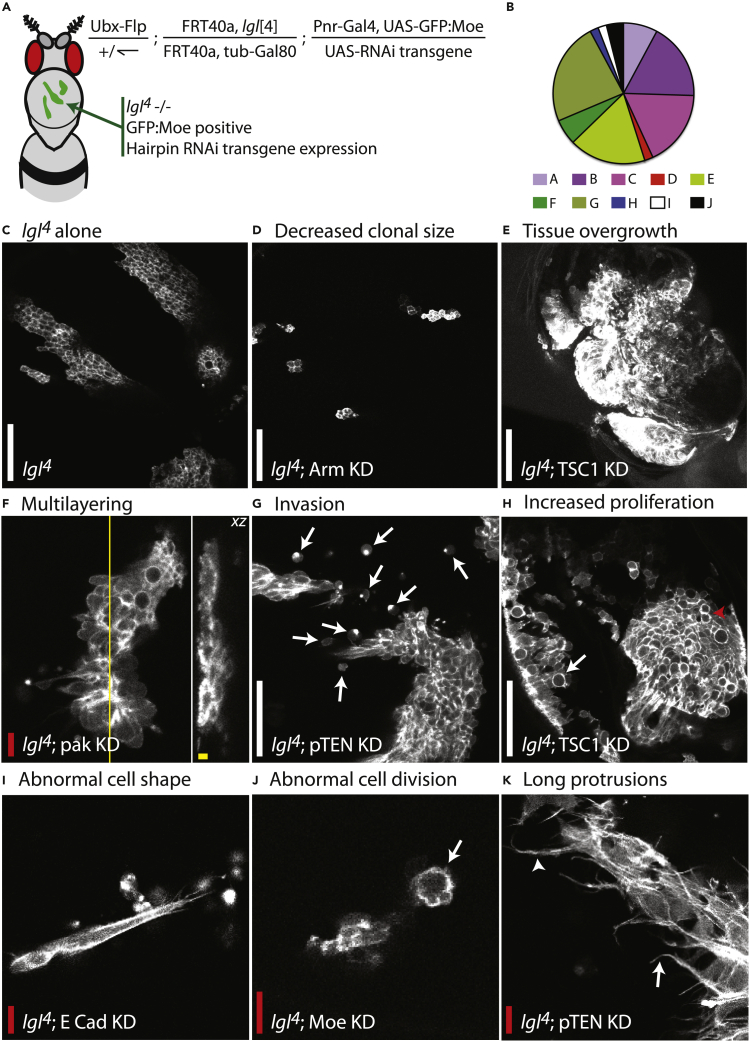
Pilot Screen Identifies Several Modulators of Tumor Behavior (A) Schematic illustrating how clones with distinct genotypes were generated on the back of the fly. The MARCM system was employed to generate mutant clones specifically within the fly dorsal thorax, through the use of Ubx-Flp. This generated GFP:Moe-labeled *lgl*^*4*^ homozygous mutant clones. RNAi transgene expression, and therefore gene KD, was restricted to the labeled *lgl*^*4*^ mutant tissue. (B) Pie chart illustrating the range of biological functions from those genes included in the pilot screen. (A) apicobasal polarity, (B) cell adhesion, (C) cytoskeleton, (D) axon guidance, (E) cell cycle, (F) gene expression, (G) signaling, (H) mitochondria, (I) others, (J) unknown. (C–K) Examples of phenotypes observed within the pilot screen. In the pilot screen we observed effects on clonal size (D and E), tissue morphology (E and F), cell morphology (I and K), and cell behavior (G, H, and J). These are just a few examples of the many distinct phenotypes that we observed. Panel (C) shows *lgl*^*4*^ clones for comparison. Arrows: (G) invading cells, (H) dividing cells, (J) a blebbing dividing cell, and (K) very long basal protrusions. Arrowheads: (H) cell doublet following cytokinesis and (K) long protrusions joining to form a fascicle. White scale bar, 50 μm; red scale bar, 10 μm; yellow scale bar, 10 μm in *xz* plane.

During an initial pilot screen, candidate genes previously implicated in cancer were studied. These genes were well characterized and therefore were very likely to present a phenotype. Also included were negative controls, i.e., RNAi lines to genes that are not normally expressed in this tissue. We used transgenic UAS-RNAi lines, which together with pannier-Gal4 and MARCM allowed us to restrict gene knockdown (KD) to *lgl*^*4*^ mutant tissue on the notum of the fly (Figure 2A). We used RNAi lines from two near-genome-wide RNAi libraries (VDRC, Austria, and NIG, Japan) and where possible used two independent RNAi transgenes to knock down gene expression for each gene. In total, the pilot consisted of 67 RNAi lines targeting 46 well-known genes (see Table S1 for a list of pilot genes). These candidates included various oncogenes, tumor suppressor genes, matrix metalloproteinases, and regulators of cell morphogenesis, with a range of biological functions (Figure 2B).

We observed a wide range of phenotypes in the pilot screen including hyperproliferation, multilayering, invasion, and effects on subcellular structures (junctions, microvilli, basal protrusions; Figures 2C–2K). Negative controls failed to generate significant phenotypes. We saw a range of expected phenotypes, for example, increased clonal coverage following RNAi of the known tumor suppressor, Tsc1 (a negative regulator of Tor signaling); reduced clonal coverage following RNAi of a known promoter of the cell cycle, tkv (promotes Dpp signaling); increased multilayering following RNAi of the polarity determinants scrib, expanded, and dlg; and smaller apices following RNAi of Cdc42, as has been observed previously (Georgiou et al., 2008) (Table S1).

Following the successful completion of the pilot screen, we went on to screen a total of 764 RNAi lines corresponding to 497 individual genes. Recent advances in genome characterization technologies have uncovered a plethora of candidate genes across numerous tumor types that have been found to be commonly mutated or misregulated in human cancers (Beroukhim et al., 2010, International Cancer Genome et al., 2010, Lawrence et al., 2014). However, other than being implicated by these new technologies, many are completely uncharacterized. By screening *Drosophila* orthologs of these previously implicated cancer genes we sought to determine which of these genes affect tumor behavior and drive tumor progression in our system.

### Systematic High-Throughput Scoring and Quality Control

We generated a database, whereby we could systematically score specific aspects of tumor behavior, allowing us to record an extremely detailed analysis of how each gene KD affected tumor behavior (see Table S1 for full database). This database consists of 33 phenotypic categories where each animal with *lgl*^*4*^ + RNAi KD clones is scored relative to animals with *lgl*^*4*^ clones alone. Each category describes an aspect of tumor behavior. Categories include clone size and shape, number of dividing cells, number of invading cells, apex size, junction defects, cytoskeletal defects, multilayering, etc. The scoring system we employed reflected the fact that gene KD could either positively or negatively affect specific aspects of tumor behavior (Figure S1). A minimum of five animals were analyzed per gene KD, and each animal was scored blind by two researchers. An online searchable database with all results from the screen, including all high-resolution images for each RNAi line, is available at https://flycancerscreen.nottingham.ac.uk.

To verify that our high-throughput qualitative scoring system gave meaningful results that represented real changes in tumor behavior, we performed a careful quantitative analysis on a selection of genes chosen at random for categories that were amenable to a simple quantitative analysis. As shown in Figures S2A–S2D, a strong positive correlation was observed for all categories measured (0.91–0.97, Spearman correlation test). To further evaluate the quality of our dataset, we asked whether two independently generated RNAi lines targeting the same gene produced similar phenotypes. We compared scores across categories for each pair of RNAi lines and found that, of the 256 genes that were targeted by two independent RNAi lines, 224 (87.5%) gave statistically similar phenotypes (Figures S2E–S2J; Table S2).

### Identification of Genes that Affect Tumor Behavior

We used an unbiased approach to identify candidate genes that increase or decrease specific aspects of tumor progression in our system. We calculated a mean score for each of the 764 RNAi lines across each of the 33 phenotypic categories (see https://flycancerscreen.nottingham.ac.uk). Using these averages, we determined the distribution of scores for all 33 categories. Genes with a mean score above or below the interquartile range from the median were selected as genes of interest. For categories with a two-tailed distribution we were able to identify genes that when knocked down either positively or negatively regulate a specific aspect of tumor behavior. For example, using this methodology we identified 66 RNAi lines that promote and 49 RNAi lines that inhibit cancer cell invasion (mean scores range from +0.73 to +1.5, and −0.55 to −1.2, respectively). See Table S3 for a full list of hits for all categories.

**Figure 3:**
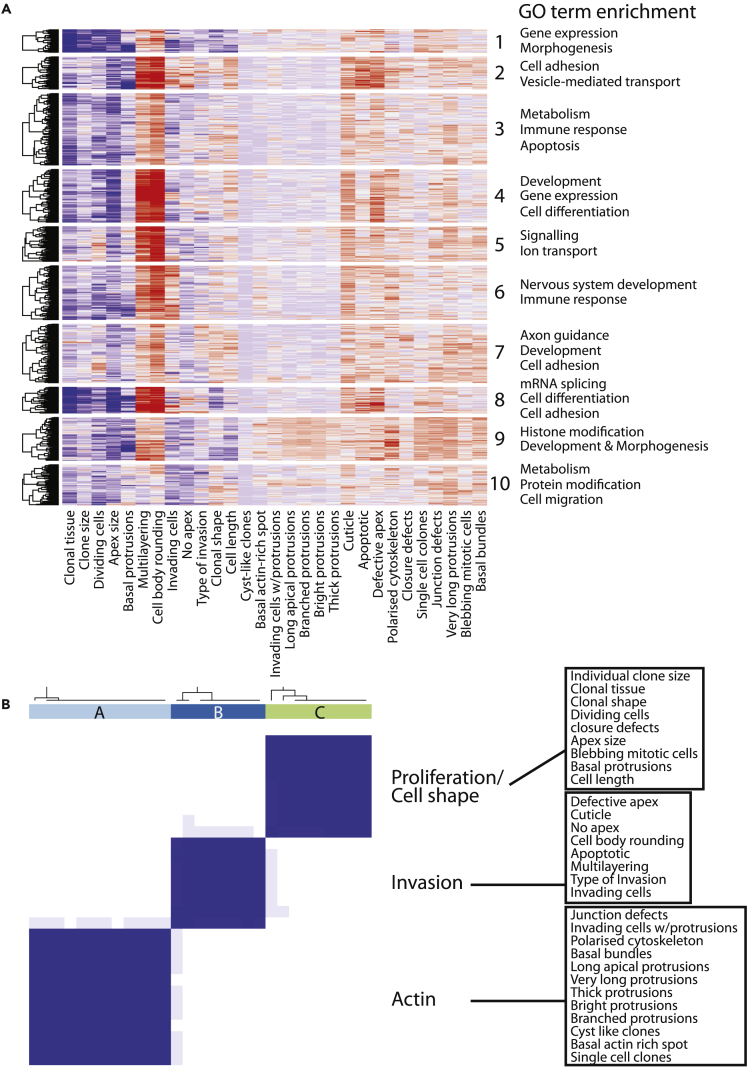
Clustering Analyses Identify 10 RNAi Line Clusters and Three Distinct Phenotypic Subgroups (A) Heatmap representation of supervised clustering of 764 RNAi lines with average phenotype scores. Each row represents an RNAi line; each column represents a phenotype category. *A priori*, the model-based optimal number of *K* = 10 (phenotypic clusters) was determined. The clustering of rows and columns are based on Euclidean distance. Map colors represent row-scaled average scores: blue indicates the lowest score, light blue indicates an intermediate score, and red indicates the highest score. Each cluster was analyzed with regard to biological function by GO enrichment analysis. The most enriched representative GO categories are shown on the right-hand side of each cluster. (B) Consensus clustering of average scores of 29 phenotypic categories reveals three distinct subgroups. Each column represents one phenotype. Heatmaps display consensus values between pairs of phenotypes by blue shading. High consensus corresponds to phenotypes that always occur in the same cluster and is shaded dark blue. See also Table S4 and Figure S3.

To identify genes that regulate similar or related cell behaviors, we clustered RNAi lines based on phenotypes presented across all categories. This resulted in the identification of 10 phenotypic clusters (Figure 3A). Analysis of the hierarchical clustering revealed, for example, that Cluster 8 shows decreased clonal tissue and increased tissue multilayering and cell body rounding (Figure 3A). Gene ontology (GO) term analysis shows enrichment in junction assembly, cell adhesion, cell differentiation, and fate specification factors (Table S4). A more general categorization of gene function reveals an increase in apicobasal polarity and cell adhesion factors (Figure S3). Therefore, Cluster 8 includes factors that are crucial to the maintenance of an ordered, monolayered, and polarized epithelium. Thus, cluster analysis reveals groups of genes with similar overall phenotypes that may share similar or related molecular functions. Within these groups lie several uncharacterized genes that we can now classify as tumor suppressors.

**Figure 4:**
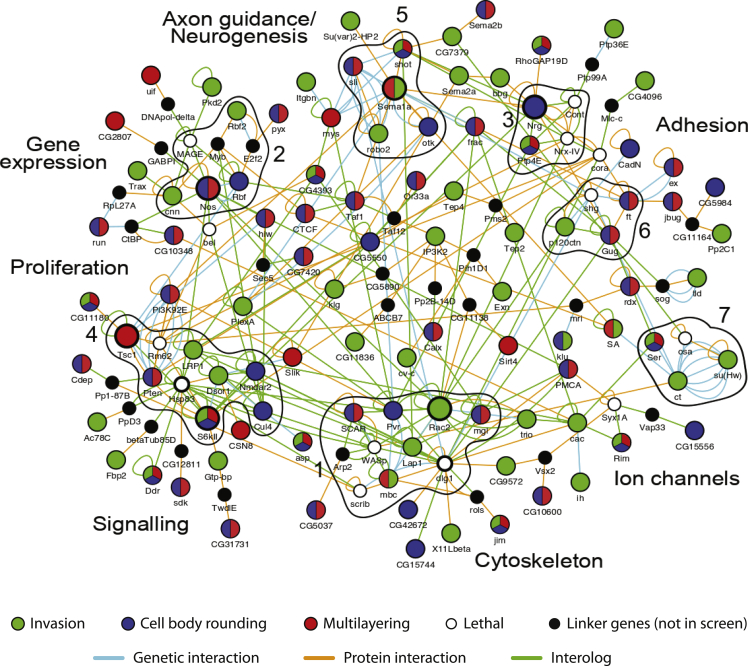
An Interaction Network of Invasion Suppressors Interactions between genes for which KD enhanced the categories “invasion,” “multilayering,” and “cell body rounding” are shown. Each circle node represents a gene. Node color indicates phenotype observed in the screen: green, invasion; blue, cell-body rounding; red, multilayering; multi-coloured nodes, genes that were hits for more than one phenotype; white, lethal; black, “linker genes,” i.e., genes that were not part of the screen, but that connect screen hit genes by one interaction; nodes with a bold outline, hub genes in this network. Lines represent interactions: cyan, genetic; orange, protein-protein; green, interolog. MCODE complexes of highly interconnected genes are outlined in black. Significantly enriched GO terms are indicated. See also Data S1.

We additionally clustered categories based on phenotypes presented across all RNAi lines and identified three distinct category clusters (Figure 3B). Categories that clustered together included those related to (A) actin cytoskeleton regulation, (B) invasion and multilayering, and (C) cell proliferation and cell and tissue morphology. We were particularly interested in the identification of novel genes that promote cancer cell invasion. Interaction networks have become a powerful tool to identify novel disease-associated genes (Sevimoglu and Arga, 2014). To generate a functionally validated interaction map of invasive genes, we combined all hits in three categories that clustered strongly together (Figure 3B): invasion, multilayering, and cell body rounding. For each gene, we searched for physical or genetic interactions, validated by experimental data, including yeast two-hybrid, co-immunoprecipitation, and other interaction data from various databases (see Methods). We maintained interactions only between hit genes from these categories, together with lethals and “linker genes,” which linked hit genes from our screen by one interaction (Figure 4; Data S1). The resulting network includes 321 interactions between 140 genes, 99 of which have not been previously implicated in cancer cell invasion or migration, including nine genes that are completely uncharacterized.

Using MCODE (Molecular Complex Detection) software (Bader and Hogue, 2003) we found seven clusters of highly interconnected nodes (Figure 4). Complex 1 comprises core proteins involved in cytoskeleton organization, including Rac2, Scar, WASp, Arp2, and mbc. Adhesion proteins highly involved in cancer invasion are present in Complex 6; Complex 5 is enriched in axon guidance molecules, whereas other identified complexes are enriched in proteins that have not been previously linked to cancer cell invasion, such as Complexes 4 and 7. By integrating hits in invasive categories from our screen, together with protein and genetic interaction data, we have therefore identified a large number of genes that are now implicated in cancer cell invasion.

### Characterization of Invading Cancer Cells

**Figure 5:**
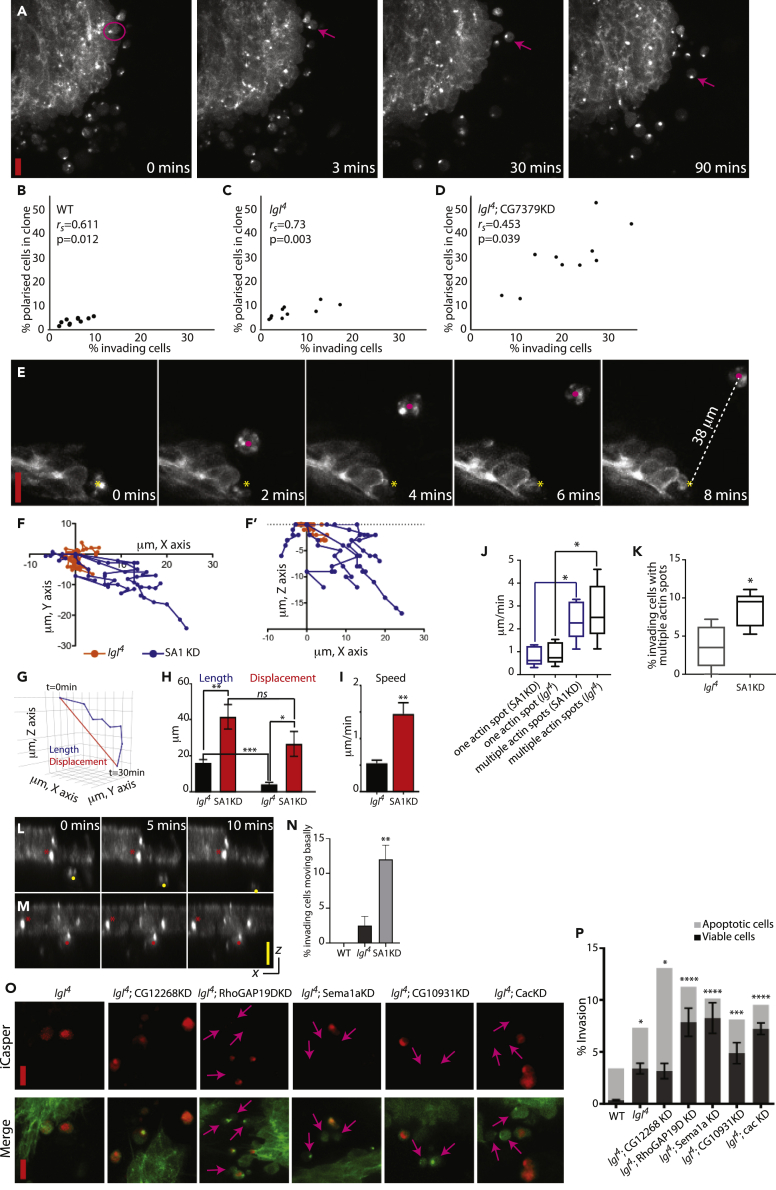
Characterization of Selected Invasion Suppressors (A) An example of a highly invasive mutant clone (genotype: *lgl*^*4*^; CG7379KD) labeled with GFP:Moe. Highlighted is a pre-invasive cell that rounds up and forms a characteristic actin-rich spot at one side of the cell before invasion (0 min). The cell then detaches from the mutant clone and migrates away (arrow). (B–D) Correlation between the percentage of clonal cells with a polarized actin accumulation and the percentage of invading cells per animal (n = 10 animals/genotype). (B) WT, (C) *lgl*^*4*^, (D) *lgl*^*4*^; CG7379KD. The two parameters show a significant correlation, irrespective of whether the mutant clones were rarely invasive or highly invasive. (E–F') (E) Stills from a time-lapse showing the basal surface of a GFP:Moe-labeled SA1KD clone. Yellow star marks the initial location of an invading cell; magenta dot shows the location of the invading cell at the indicated time. The cell shown has moved 38 μm in 8 min. (F and F′) Representative single-cell trajectories from *lgl*^*4*^ (orange) and SA1KD invading cells (blue) shown in *xy* (F) and *xz* (F′). Each cell was measured every 3 min for 30 min. (G) Illustration showing the two trajectories measured for each invading cell to determine directionality. Length (blue) follows the full trajectory of an invading cell. Displacement (red) measures a straight line from the initial to the final point. (H) Quantification of length and displacement from *lgl*^*4*^ and SA1KD cells (n = 25 cells from 5 animals/genotype). Cells that have directionality have no significant difference between length and displacement. (I) Quantification of speed of migration, showing average micrometers traveled per minute (n = 25 cells from 5 animals/genotype). (J) Quantification of speed of migration (μm/minute) for *lgl*^*4*^ and SA1KD cells that present either a single actin spot or multiple actin spots (n = 5 cells/group). Those with multiple spots travel faster irrespective of genotype. (K) SA1KD cells have a significantly higher proportion of invading cells with multiple actin spots (n = 5 animals/genotype). (L and M) Orthogonal view of invading cells showing that cells only migrate once detached from the epithelial sheet (yellow dot, (L)). Red asterisk, pre-invasive cell within sheet; red dot, delaminated cell still attached to sheet (M). (N) Quantification of the percentage of pre-invasive cells that detach from the epithelial sheet and migrate, in WT, *lgl*^*4*^, and SA1KD clones (n = 3 animals/genotype). (O) iCasper (red) and GFP:Moe (green)-labeled mutant clones (genotypes specified above panels). Arrows highlight invading cells that are iCasper negative. (P) Four of the five invasive genotypes tested showed a high proportion of invading cells that were iCasper negative (n = 10 animals/genotype). Error bars = ± SEM. Student's t test or one-way ANOVA with Dunnett's post hoc test for multiple comparisons was performed to determine statistical significance. p > 0.05 was considered not significant, ∗p < 0.05, ∗∗p < 0.01, ∗∗∗p < 0.001, ∗∗∗∗p < 0.0001. Red scale bar, 10 μm; yellow scale bar, 10 μm in the *xz* plane. See also Figure S4.

With the aim of characterizing the behavior of individual invading cells, we followed cells within mutant clones over time, before, during, and post-invasion. We found, in all genotypes studied, that pre-invasive cells would round up and form a characteristic actin-rich spot at one side of the cell before invasion (Figure 5A, Video S1). By calculating the coefficient of determination using Spearman's rho (*r*_*s*_) we observed a high-to-moderate positive correlation between a polarized actin accumulation and invasion in all genotypes studied, irrespective of whether the mutant clones were rarely invasive or highly invasive (Figures 5B–5D). The number of cells presenting this polarized phenotype within the epithelial sheet is therefore an indicator of invasive potential.

Video S1. Non-directional Migration Time-lapse movie of a highly invasive mutant clone (genotype: *lgl*^*4*^; CG7379KD) labeled with GFP:Moe, showing invading cells with non-directional migration. Time stamp: top left; scale bar, 10 μm. See also Figure 5

A major advantage of our *in vivo* model is that the directionality and speed of invading cells can be studied and quantified in real time (Figures 5A–5I). It was notable that in many cases, invading cells, although viable, have no directionality to their migration and randomly move about over a number of hours (Figure 5A, Video S1). However, in some cases, as in the case of SA1KD, invading cells appear to be very motile (Figures 5E–5I, Video S2). Single-cell tracking of *lgl*^4^ and SA1KD-invading cells was performed to determine the X, Y, and Z trajectories and to calculate their speed and directionality. An illustration of representative trajectories is shown in Figures 5F and 5F′. To determine directionality, the trajectory of each cell was measured over 30 min. The total number of micrometres traveled was documented (length in Figures 5G and 5H) as well as the distance an invading cell would have traveled if following a straight line (displacement in Figures 5G and 5H). Figure 5H shows a significant increase in length and displacement for SA1KD cells (41.55 μm length, p < 0.01; 26.55 μm displacement, p < 0.05) when compared with *lgl*^*4*^ cells (16.07 μm length; 4.16 μm displacement). There is no significant difference between length and displacement in SA1KD cells, indicating that their trajectories are directional. In addition, the speed of migration for SA1KD cells was 2.7-fold higher (1.46 μm/min, p < 0.01) when compared with *lgl*^*4*^-invading cells (0.53 μm/min; Figure 5I). It also became apparent that those cells that migrated in a fast, directional fashion did not possess a single actin-rich spot, but multiple dynamic actin-rich spots (Figure 5E), and quantification of migrating cells showed that those cells with multiple spots migrated at a significantly faster rate. We additionally found that a low proportion of *lgl*^*4*^-invading cells can possess multiple actin-rich spots, which also migrate in a directional fashion (Figures 5J and 5K), indicating that this change in cytoskeletal organization and behavior is important to promote directional migration, irrespective of mutant background.

Video S2. Directional Migration Time-lapse movie of an SA1KD clone labeled with GFP:Moe, showing invading cells with fast, directional migration. Time stamp: top left; scale bar, 10 μm. See also Figure 5

When imaging pre-invasive and invading cells in the *xz* plane, we found that cells that are still attached to, or within, the epithelial sheet show very limited lateral movement, and only migrate once they are fully detached from the sheet (Figures 5L and 5M). We additionally found that invading cells detach from the epithelial sheet more readily in SA1KD clones than in *lgl*^*4*^ clones, which corresponds with SA1KD clones being highly invasive, with invading cells that exhibit directional migration (Figure 5N).

It has previously been shown that WT epithelial cells delaminate from the pupal notum at early pupal stages, but this delamination is concentrated at the midline region and is rapidly followed by cell death (Fujisawa et al., 2019, Marinari et al., 2012). This is in stark contrast to the behavior of invading cells within highly invasive tumors in our screen, where invasion is observed irrespective of the clone's position within the epithelial sheet, and invading cells do not undergo immediate cell death (we have imaged invading cells for up to 2 h without observing cell death; for example, see Figure 5A and Video S1). To specifically test for the viability of invading cells within highly invasive tumors, we used the genetically encoded apoptosis reporter iCasper (To et al., 2015). We expressed iCasper within WT clones, *lgl*^*4*^ clones, and in clones for five strong hits for invasion from our screen, namely, *lgl*^*4*^; CG12268KD, *lgl*^*4*^; RhoGAP19DKD, *lgl*^*4*^; Sema1aKD, *lgl*^*4*^; CG10931KD, *lgl*^*4*^; CacKD. We observed that in four of the five invasive genotypes tested, a high proportion (∼70%) of invading cells were iCasper negative. Only WT, *lgl*^*4*^ alone, and *lgl*^*4*^; CG12268KD mutant clones showed a high proportion of invading cells that were positive for apoptosis (∼64%; Figures 5O and 5P).

Having identified a number of invasion suppressors in our screen, we wanted to test whether human orthologs of the fly genes within this category would also act in a similar way. We took a panel of five fly genes that (1) strongly promote invasion when their expression is knocked down, and (2) have high-confidence, high-scoring best match human orthologs (Hu et al., 2011). Genes included were RhoGAP19D, Rim, S6kII, CG7379, and shot (their closest human orthologs are ARHGAP23, RIMS2, RPS6KA3, ING1, and DST). We designed small interfering RNAs (siRNAs) against these human genes to see if their loss would lead to similar effects in the MCF7 breast cancer cell line. We used an *in vitro* invasion assay to test whether gene KD would promote MCF7 invasion and/or migration. We found a significant increase in both invasion and migration following gene KD of RPS6KA3, ING1, and DST, and a significant increase in migration alone with gene KD of RIMS2 (Figure S4).

These results provide strong evidence that our *in vivo* system can identify regulators of tumor progression and cancer cell invasion. Results show that in most cases invading cells are non-apoptotic, and that this model can provide additional insight on invading cell morphology and behavior, which can indicate a tumor's invasive potential. Results also suggest that the invasion hits identified in our genetic screen are likely to have relevance to human disease.

### The Cohesin Complex Is an Invasion Suppressor

**Figure 6:**
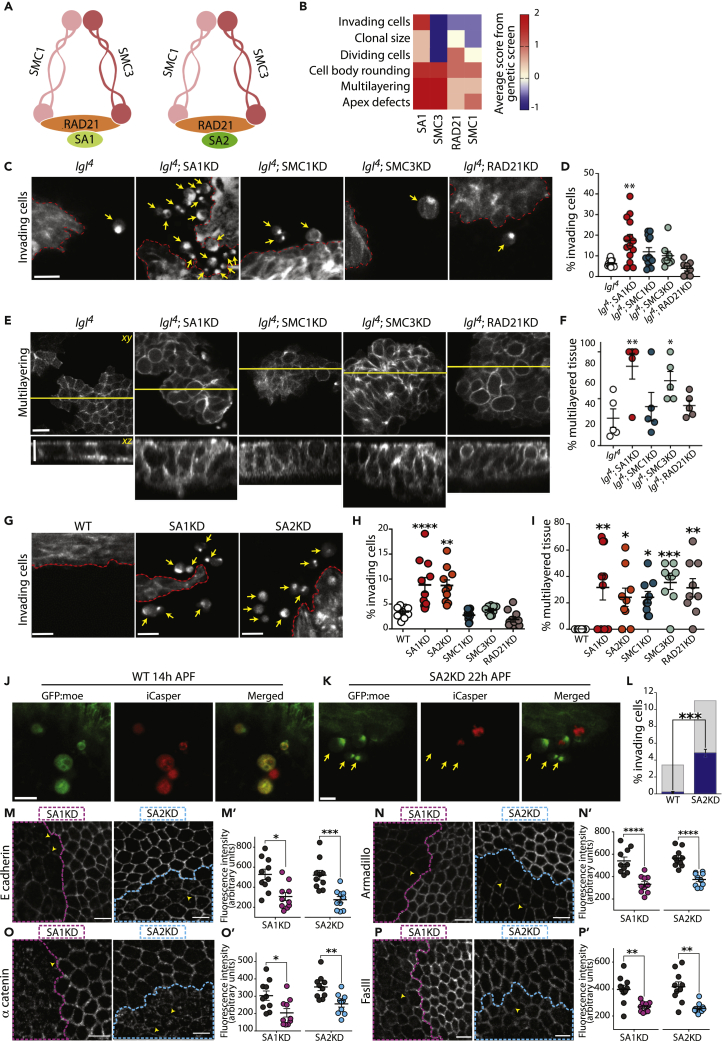
SA1 or SA2KD Promotes Invasion (A) Somatic cells simultaneously express two different Cohesin rings, differentiated by the presence of either SA1/STAG1 or SA2/STAG2. (B) Heatmap illustrating qualitative scores given to cohesin subunits included in the genetic screen. A subset of categories is shown. Red, enhancement of a phenotype; yellow, no phenotype change; blue, inhibition of a phenotype. (C–F) GFP:moe positively marked *lgl*^*4*^ mutant clones with additional cohesin complex subunit KD, showing invading cells (arrows; (C)) and multilayering (E), quantified in (D) and (F); n = 5 animals/genotype. Red dashed line highlights edge of clone. Yellow line shows position of *xz* slice shown. (G) Basal confocal slice of GFP:moe positively marked WT, SA1, or SA2KD clones, highlighting invading cells (arrows). (H and I) Quantification of % invading cells (H) and % multilayering (I) following KD of each cohesin subunit, compared with WT. (J–L) Confocal images of the basal surface of iCasper (red) and GFP:Moe (green)-labeled WT clones (J) and SA2KD clones (K). Arrows highlight invading cells that are iCasper negative. Quantified in (L): Gray, % invading cells/total number of labeled cells; blue, % non-apoptotic invading cells/total number of labeled cells; n = 50 cells from 10 animals/genotype. Young WT pupae were used as a control (J) as older WT animals have little to no invading cells. (M–P) SA1 or SA2KD clones, highlighted by magenta and cyan dashed lines, respectively, show disrupted E-cadherin (M, quantified in M'), armadillo (N, quantified in N'), α-catenin (O, quantified in O'), and fasIII (P, quantified in P'), localization. Arrowheads highlight junctional breaks. Quantification shows fluorescence intensity at the level of the junction (n = 100 junctions from 10 animals for each genotype). Scale bars, 10 μm. Error bars = ± SEM. Student's t test or one-way ANOVA with Dunnett's post hoc test for multiple comparisons was performed to determine statistical significance. p > 0.05 was considered not significant, ∗p < 0.05, ∗∗p < 0.01, ∗∗∗p < 0.001, ∗∗∗∗p < 0.0001. See also Figures S5 and S6.

Cohesin is a multi-protein complex that forms a tripartite ring-like structure consisting of the proteins SMC1, SMC3, and RAD21 (Gruber et al., 2003). In addition, RAD21 binds to a stromalin protein (SA1 or SA2, also known as STAG1 or 2 in humans) (Losada et al., 2000, Zhang et al., 2013) (Figure 6A). Therefore two cohesin complexes can form, with cohesin genomic distribution subject to a great degree on the SA/STAG protein that binds to the tripartite ring (Kojic et al., 2018). Cohesin is evolutionarily conserved, with functional cohesin complexes found ubiquitously in all eukaryotic organisms, from yeast to humans (Michaelis et al., 1997, Losada et al., 2000). The cohesin complex is mainly known for its role in sister chromatid cohesion (SCC) (Michaelis et al., 1997); however, current understanding of the possible and numerous roles cohesin may play in tumor initiation and cancer progression is limited (de Koninck and Losada, 2016).

Four subunits of the cohesin complex were studied in our genetic screen: SMC1, SMC3, RAD21, and SA1. Knockdown of these subunits induced significant cytoskeletal changes to *lgl*^*4*^ tumors, including increased multilayering, cell body rounding, and apex defects. In addition, SA1KD significantly enhanced the *lgl*^*4*^ invasive phenotype, with other cohesin subunits having no effect on invasion (Figures 6B–6F). We next knocked down the expression of specific cohesin subunits in WT clones and found that SA1 and SA2KD strongly promoted invasion even in the absence of the *lgl*^*4*^ mutation, whereas the other subunits did not; all subunits, however, promoted multilayering (Figures 6G–6I). Using iCasper we also saw that a high proportion of invading cells evaded apoptosis (Figures 6J–6L) and as shown earlier, showed fast directional migration (Figures 5E–5K; Video S2).

Our screen identified cohesin subunits as affecting epithelial architecture, cell shape, and in the case of SA subunits, promoting frequent cell delamination. These phenotypes therefore implicate effects on adhesion, polarity, and actin regulation as possible underlying influences on the observed cell behavior. We investigated cell-cell adhesion and polarity using antibodies to proteins that localize to the adherens junction (AJ), septate junction, and the sub-apical region. We generated SA1 and SA2KD clones and directly compared junction composition inside and outside the clones within the same tissue. A significant reduction in the cortical localization of E-cadherin, α-catenin, β-catenin, and FasIII was observed at the junctional level in both SA1 and SA2KD clones, when compared with the surrounding WT tissue, with evidence of junctional breaks, ectopic structures (puncta, tubules), and mislocalization of junction components (Figures 6M–6P), which are phenotypes that are commonly observed when junctional integrity is compromised (Georgiou et al., 2008). In contrast, KD had no effect on the polarity proteins investigated (dlg and aPKC; Figure S5). These results suggest that SA1 and SA2 act as invasion suppressors in part through the correct localization of junction determinants, thereby maintaining cell-cell junction integrity.

To determine if the role of SA1 and SA2 as invasion suppressors is conserved, we next studied the effect that the loss of their human orthologs, STAG1 and STAG2, would have on MCF7 cell invasion and migration using an *in vitro* invasion assay. LOF mutations of *STAG2* are significantly elevated in metastatic breast cancer tumors when compared with lower grades (Repo et al., 2016), suggesting that *STAG2* has a role in preventing tumor transition to malignancy. *STAG2* is also commonly mutated in several cancer types, including bladder cancer and Ewing sarcoma (Aquila et al., 2018, Tirode et al., 2014). When analyzing each cohesin subunit in turn we found that only STAG1 and STAG2KD promoted invasion and migration, with the core components of the tripartite ring failing to affect cell behavior (Figures S6A–S6I) thereby mirroring the effect we see *in vivo* in the fly (Figures 6G and 6H).

Cohesin is known to influence gene expression. It has been shown in yeast and flies that substantial reductions in cohesin dosage of more than 85% are required to disrupt cohesion and chromosome segregation, whereas small to moderate reductions can affect gene expression (Dorsett and Merkenschlager, 2013). Therefore, the invasive effects that we see in SA/STAG mutants could be due to changes in the expression of genes that affect cell-cell junctions and/or the cytoskeleton. As STAG2 is the most abundant and most mutated cohesin gene in human cancers we performed a microarray gene expression analysis, comparing gene expression in MCF-7 cells post STAG2KD with untreated cells (unt) and with cells treated with non-targeting siRNA (non-T). Of 21,448 genes analyzed, the expression of 23 genes was significantly altered as a result of STAG2KD (p < 0.01, fold change [FC] ≥ 1.5 or FC ≤ −1.5; Figure S6 and Table S5). We additionally used RT-qPCR on a selection of genes (STAG2, PCDH1, EHD2 and AKR1B10) to verify the microarray results, with qPCR showing the same or stronger expression change in all cases (Figure S6N).

GO term analysis identified six biological processes that were significantly enriched within the 23 differentially expressed genes, including cell-cell adhesion, protein localization, and cell projection organization (Figure S6O). In addition, an interaction network was generated, using the Cytoscape plugin GeneMania, to display any genetic and physical interactions, verified by experimental data, between the differentially expressed genes and members of the AJ KEGG pathway (Figure S6P; Data S2). Ninety-five interactions between 20 differentially expressed genes and 20 AJ KEGG pathway genes indicate that the differentially expressed genes in STAG2KD cells extensively interact with members of the AJ pathway. Furthermore, EHD2 was significantly downregulated in STAG2KD cells. EHD2 has been linked to E-cadherin localization and expression, and lower EHD2 expression is associated with metastatic tumors (Shi et al., 2015, Yang et al., 2015). EHD2 links endocytosis to the actin cytoskeleton (Guilherme et al., 2004) and could therefore be influencing E-cadherin's ability to recycle at the junction.

An additional GO term analysis was performed on differentially expressed genes found in two studies that depleted STAG2 expression in cell lines of epithelial origin (MCF10A, Kojic et al., 2018, and HCT116, Casa et al., 2020). Here we found statistically enriched terms including regulation of cell-cell adhesion, regulation of cellular protein localization, regulation of cell-matrix adhesion (Kojic et al., 2018), and positive regulation of cell migration (Casa et al., 2020).

### Cohesin Loss of Function Induces the Formation of a Supracellular Actomyosin Ring

**Figure 7:**
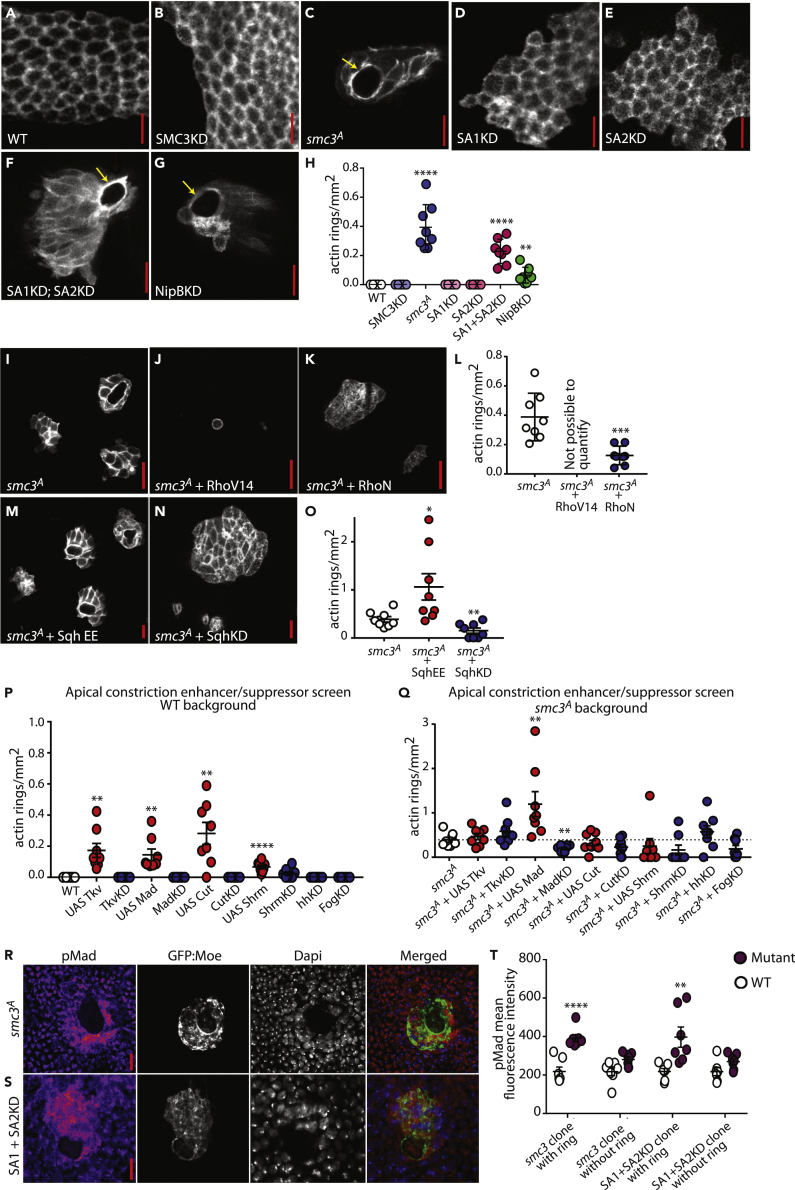
A More Severe Cohesin LOF Induces Actin Ring Formation (A–G) GFP:moe positively marked WT (A), SMC3KD (B), *smc3*^*A*^(C), SA1KD (D), SA2KD (E), SA1KD; SA2KD (F), and NipBKD (G) clones. Actin rich rings (yellow arrows) were observed in *smc3*^*A*^, SA1, and SA2 simultaneous KD and NipBKD clones. (H) Quantification of the number of actin rings per square millimeter of clonal tissue. Eight animals were analyzed for each genotype. (I–O) GFP:moe positively marked clones ((I) *smc3*^*A*^; (J) *smc3*^*A*^ + RhoV14; (K) *smc3*^*A*^ + RhoN; (M) *smc3*^*A*^ + Sqh EE; (N) *smc3*^*A*^ + SqhKD). Dominant-negative Rho (RhoN) and SqhKD inhibit actin ring formation in *smc3*^*A*^ clones; phosphomimetic Sqh (SqhEE) increases the number of clones with actin rings. Quantified in (L) and (O) showing the number of actin rings or delaminated clones per square millimeter of clonal tissue. Each dot represents one animal. *smc3*^*A*^ + RhoV14 resulted in very small unicellular clones (J) or no clones at all and could not be quantified. (P and Q) Genes involved in apical constriction were either knocked down or overexpressed in GFP:moe positively marked clones, either on their own (P) or within *smc3*^*A*^ clones (Q). Quantification shows the number of actin rings or delaminated clones per square millimeter of clonal tissue. Each dot represents 1 animal. (R and S) GFP:moe-labeled *smc3*^*A*^ (R) and SA1 + SA2KD (S) clones stained for the active form of the Dpp signaling effector, phosphorylated Mad (pMad). (T) Quantification of mean fluorescence intensity from the nuclei of cells within clones, with and without actin rings, compared with WT tissue within the same animal. 35 nuclei from 7 animals were measured. Each dot represents one animal. Scale bars, 10 μm. Error bars = ± SEM. Statistical analysis: Student's t test. p > 0.05 was considered not significant, ∗p < 0.05, ∗∗p < 0.01, ∗∗∗p < 0.001, ∗∗∗∗p < 0.0001. See also Figure S7.

Although SA1KD, SA2KD, and SMC3KD promote multilayering (Figure 6I), at an apical level they present a phenotype very similar to WT, with cells presenting an organized geometric shape (Figures 7A, 7B, 7D, and 7E). By contrast, we see a very different phenotype for three cohesin LOF genotypes: *smc3*^*A*^ (an ethyl methane sulfonate-induced truncating mutation within *smc3*, K575term, Haelterman et al., 2014, Yamamoto et al., 2014); combined SA1 + SA2KD; and NipBKD (loss of NippedB prevents cohesin from interacting with DNA, Ciosk et al., 2000). These mutants induced a highly distinctive phenotype with drastic cytoskeletal changes, including the formation of a supracellular actin ring (Figures 7C and 7F–7H), eventually followed by clonal extrusion (Figure S7C). It therefore appears that a more severe disruption to cohesin function leads to a very different phenotype to that observed when a single SA subunit is KD. Here individual cell invasion is not observed, rather apical constriction and basal clonal extrusion occur, which are likely to have relevance to the poorly understood process of collective cell invasion in cancer. We further characterized the phenotype using both GFP:Moe to label actin and mCherry:spaghetti squash (sqh; the fly ortholog of the regulatory light chain of non-muscle myosin II). We found that the supracellular ring is enriched with actomyosin, which induces the invagination of the mutant tissue, forming a ball of cells with a central lumen (Figures S7B–S7D). We also found significantly elevated levels of E-cadherin within *smc3*^*A*^ clones (Figures S7D and S7F), which could also promote clonal invagination through differential adhesion properties between cell types (Steinberg, 2007).

Long time-lapse movies show that over a number of hours the actomyosin ring contracts, inducing a basal clonal extrusion from the epithelial sheet (Figure S7C). Using the caspase sensor, iCasper, we found no significant difference in the levels of apoptosis in *smc3*^*A*^ clones, irrespective of whether the clone was still connected to the epithelial sheet or had already extruded (Figure S7G). Furthermore, time-lapse imaging was performed on extruded clones with little increase in iCasper signal observed over 1 h post-extrusion (Figure S7H), indicating that the basal extrusion of *smc3*^*A*^ clones does not trigger extensive cell death.

Known mechanisms that trigger apical constriction during development include the apical localization of activated Rho1, which recruits and activates myosin II (Padash Barmchi et al., 2005). We found that Rho1 and Sqh are essential for the determination of *smc3*^*A*^ cell morphology and actin ring formation, as dominant-negative Rho (RhoN) and SqhKD both inhibit actin ring formation and clonal extrusion, whereas phosphomimetic Sqh (Sqh-EE) significantly increases the prevalence of this phenotype (Figures 7I–7O).

To better understand the potential mechanism of action of SMC3 in apical constriction and actin ring formation, an enhancer/suppressor screen of genes involved in regulating the localization of myosin II and Rho1 to the apex of the cell was performed. Six candidate genes were KD and, where possible, overexpressed, both alone and in combination with the *smc3* mutation, to determine if these genes enhance or rescue the actin ring and clonal extrusion phenotype. Although four genes promoted actin ring formation in WT clones when overexpressed, only Mad had any significant effect within *smc3*^*A*^ clones. Mad overexpression within *smc3*^*A*^ clones significantly increased the number of actin rings and delaminated clones (1.196, n = 8, p < 0.05) when compared with *smc3*^*A*^ alone (0.393, n = 8), whereas MadKD in *smc3*^*A*^ tissue had the opposite effect (0.196, n = 8, p < 0.01; Figures 7P and 7Q).

Mad is the main effector of the *Drosophila* Dpp signaling pathway. An increase in Dpp signaling has been directly implicated in apical constriction and actin ring formation (Jidigam et al., 2015). Using a phospho-Mad antibody (pMad) we detected a significant increase in pMad levels in *smc3*^*A*^ clones and SA1 + SA2KD clones, specifically when these clones contained actin rings (Figures 7R–7T) suggesting that an increase in Mad activity is necessary to induce apical constriction in cohesin LOF clones. It therefore appears that an upregulation of Dpp signaling is a key determinant for the collective invasion observed in cohesin LOF clones.

Given the known pleiotropic effects of the cohesin complex (on SCC, homologous recombination, genome organization and gene transcription, among others) and given our findings showing that cohesin subunits can regulate individual or collective cell invasion in an apparent dose-dependent manner, we studied the dynamics of chromosomal architecture in dividing cells *in vivo*. We generated WT, *smc3*^*A*^, SA1KD, and SA2KD clones, which were labeled with both GFP:Moe and Histone:RFP and carried out live imaging of dividing cells within these clones. We found the vast majority of *smc3*^*A*^ mutant cell divisions were defective in chromosome alignment and/or chromosome separation during metaphase and anaphase, respectively. In contrast, the vast majority of divisions in SA1 and SA2KD cells appeared normal (Figure S8; Videos S3, S4, S5, and S6) adding to the growing body of evidence to suggest that only a major reduction of cohesin function leads to cohesion and segregation defects (de Koninck and Losada, 2016).

Video S3. *In Vivo* Imaging of Cell Division, Related to Figures 6 and 7 Time-lapse movies of WT clones, labeled with GFP:Moe and Histone:RFP. Time stamp: bottom right; scale bar, 5 μm. See also Figure S8

Video S4. *In Vivo* Imaging of Cell Division, Related to Figures 6 and 7 Time-lapse movies of *smc3*^*A*^ clones, labeled with GFP:Moe and Histone:RFP. Time stamp: bottom right; scale bar, 5 μm. See also Figure S8

Video S5. *In Vivo* Imaging of Cell Division, Related to Figures 6 and 7 Time-lapse movies of SA1KD clones, labeled with GFP:Moe and Histone:RFP. Time stamp: bottom right; scale bar, 5 μm. See also Figure S8

Video S6. *In Vivo* Imaging of Cell Division, Related to Figures 6 and 7 Time-lapse movies of SA2KD clones, labeled with GFP:Moe and Histone:RFP. Time stamp: bottom right; scale bar, 5 μm. See also Figure S8

In summary, this work has (1) identified numerous genes that affect tumor behavior in a wide variety of ways; (2) generated a functionally validated network of invasion-suppressor genes; (3) identified the cohesin complex as an important invasion suppressor that can promote individual or collective invasion, dependent on severity of LOF; and (4) established the fly pupal notum as an excellent *in vivo* system to study tumor progression.

## Discussion

By combining the genetic amenability of *Drosophila melanogaster* with the power of RNAi transgenics, we were able to generate tumors with specific genotypes and to monitor tumor behavior in the living animal. The *in vivo* system we have developed offers a number of significant advantages and is particularly suitable to the study of tumor progression and invasion. It enables us to (1) monitor GFP:Moe-labeled tumors *in situ*, surrounded by WT tissue and the native local microenvironment; (2) image tumors in high spatial and temporal resolution over a number of hours or even days post-tumour induction; and (3) KD gene expression specifically within the developing tumor, allowing us to investigate the tumor promoting potential of numerous genes that would be developmentally lethal under classic mutation conditions.

Cancer genomes show extreme heterogeneity, with individual solid organ tumors possessing on average > 50 non-silent mutations in the coding regions of different genes (Greenman et al., 2007, Jones et al., 2008, Ding et al., 2008, Network, 2011). Breast and colorectal cancers have been found to be the most heterogeneous, with an average of 84 and 76 mutations per tumor, respectively (Sjoblom et al., 2006, Wood et al., 2007). Further complexity is evident when considering epigenetic alterations that can contribute to tumorigenesis and tumor progression (Jones and Baylin, 2007). The challenge is to identify those genes, from the many that have been implicated in human cancer, that drive cancer progression. We used our *in vivo* system to investigate a set of almost 500 genes, whose human orthologs have previously been implicated in cancer and have now identified numerous genes that either positively or negatively regulate specific aspects of tumor behavior within an epithelium in a living animal.

To understand tumor transition to malignancy, and to develop new therapeutic strategies, it will be key to paint a detailed picture of the complex signaling processes that occur during tumor progression. Our database incorporates 33 phenotypic categories and therefore offers a unique starting point to elucidate the molecular mechanisms of multiple aspects of tumor progression.

However, our primary focus was invasion, and our screen identified numerous genes that regulate epithelial cancer cell invasion. We generated a functionally validated network of invasive genes; GO term analysis of this network identified several terms that are significantly enriched, indicating processes that are likely to be important for invasion to take place. This includes adhesion, cytoskeletal remodeling, signaling, and intriguingly many axon guidance molecules. The Slit, Robo, and Semaphorin families have been previously implicated as both tumor and metastasis suppressors in breast cancer. SLIT/ROBO signaling has been postulated to prevent invasion by maintaining proper cell-cell adhesion, thereby inhibiting the detachment of tumor cells (Yuasa-Kawada et al., 2009). Many other axon guidance genes have been found to be invasion suppressors in our screen, as have uncharacterized genes that genetically interact with axon guidance genes, opening up an intriguing avenue of future research. It is clear that a loss of polarity and a disruption to normal adhesion are pivotal to promoting the process of invasion. Axon guidance proteins, being heavily involved in developmental processes that require cell movement, could be promoting invasive characteristics via these two fundamental processes.

Our *in vivo* system is furthermore particularly suited to imaging the invasive process. Our observation of characteristic cell shape changes (cell rounding and a polarized actin enrichment) that accompany invasion has been previously reported and associated with invasion (Sahai and Marshall, 2003, Yamazaki et al., 2005). However, an important avenue of future research will be to investigate the morphological and molecular processes that underlie the differential behavior between invading cells with and without directional migration. Cell body rounding would indicate an ameboid type migration, but the characteristic blebbing of ameboid migration is only clearly obvious in those cells undergoing directional migration. The use of a membrane (rather than actin-associated) marker together with high-resolution microscopy would help to determine whether the extent of membrane blebbing is an important attribute for directionality in this system. An additional consideration is the genetic simplicity of these tumors. It is evident that, in the fly, where there is less redundancy in key regulatory genes, we are able to generate multilayered, invasive tumors, with just two key mutations, but for many invasion suppressors further cooperative mutations are likely to be required to promote directional migration. Extracellular matrix composition and the presence or absence of a chemotactic gradient are also important considerations for directed migration and will be influencing cell behavior here (Talkenberger et al., 2017).

Our work on the cohesin complex provides an example of how specific phenotypes observed in our screen can inform downstream characterization analyses and provides further validation that our screen is picking up important regulators of tumor progression.

Cohesin was initially identified for its role in SCC in yeast (Guacci et al., 1997, Michaelis et al., 1997) and *Xenopus* (Losada et al., 1998) but has subsequently been found to be involved in homologous recombination-mediated DNA repair, higher-order chromatin structure and transcriptional regulation (Nasmyth and Haering, 2009, Mizuguchi et al., 2014, Phillips-Cremins et al., 2013, Hadjur et al., 2009, Seitan et al., 2013, Zuin et al., 2014). How cohesin performs these multiple roles is not fully understood, but is thought to be largely due to cohesin's ability to hold DNA strands in either *trans* (during cell division) or *cis* (generating chromatin loops) (de Koninck and Losada, 2016). This wide variety of functions complicates our understanding of how cohesin mutations may contribute to cancer progression. Inactivating mutations in genes that encode either the core cohesin subunits or the regulatory proteins that affect cohesin function (e.g., PDS5A/B, WAPL, CDCA5, NIPBL, MAU2, etc.) are common in numerous cancer types, including bladder, melanoma, colorectal, lung, Ewing sarcoma, and myeloid malignancies. Importantly, there is no clear correlation between the presence of cohesin mutations and aneuploidy in many tumor types, with recent studies implicating effects on chromatin structure, transcription, DNA repair, and stem cell/progenitor differentiation as important phenotypes that could promote cancer progression (Hill et al., 2016, de Koninck and Losada, 2016). Although cohesin is essential for cell viability, mutations are likely to reduce the amount of total functional cohesin within the cell, which will affect these diverse cohesin-mediated tasks in different ways, depending on the subunit that is mutated, the nature of the mutation, and the cell type affected. Our work shows that, as each specific mutation impacts cohesin function in different ways, effects on tumor cell behavior can range from defects in epithelial architecture to the promotion of either individual or collective invasion; the phenotype observed will depend on whether the mutation leads to a modification or a disruption of cohesin function, and the degree of any such disruption.

We found loss of cohesin function to induce different phenotypes related to actin cytoskeleton rearrangement. KD of one subcellular localization subunit, SA1 or SA2, increased invasion, multilayering, and apex defects. Reduced expression of the core subunits, SMC1, RAD21, and SMC3, increased multilayering and apex defects, yet had no effect on invasion. A more severe loss of cohesin function (an LOF *smc3* allele, SA1 + SA2 simultaneous KD, or NipBKD) induced clonal extrusion and collective invasion. Differences in cohesin subunit function (SA1 and SA2 provide subcellular localization; SMC1, SMC3, and RAD21 form the core of the ring) (Gruber et al., 2003) and isoform redundancy (SA1/SA2, SMC1A/SMC1B) (Losada et al., 2000, Revenkova et al., 2004), in combination with the specific dose required for each subunit to efficiently perform its role in either gene expression regulation or SCC (Laugsch et al., 2013), could be key to understanding the different effects observed in this study. Several recent studies have shown that individual loss of SA1 or SA2 has different effects compared with loss of all cohesin (Rao et al., 2017, Schwarzer et al., 2017, Wutz et al., 2017) and that the two SA subunits are not fully functionally interchangeable (Kojic et al., 2018). Therefore, loss of one specific SA subunit will have drastic effects on how cohesin interacts with chromatin and on gene expression. Our *in vivo* experiments in the fly and transcriptomics experiments *in vitro* suggest that loss of SA1 or SA2 induces single-cell invasion by affecting cohesin-mediated gene expression during interphase, with strong effects on junction stability. Our live cell imaging of SA1 and SA2KD cells provides further evidence to suggest that aneuploidy is unlikely to make a major contribution to this phenotype. By contrast, a severe loss of cohesin function due to a loss of functional SMC3 does lead to chromosomal instability, which ultimately leads to a misregulation of DPP signaling and increased E-cadherin levels, followed by clonal extrusion. This phenotype could be due to a combination of chromosomal instability, aneuploidy, and chromatin rearrangement defects.

### Limitations of the Study

One limitation of the screen, as is the case for any cancer screen, is the fact that the results presented here describe tumor behavior within a specific tissue and anatomical location (the fly notum) and against a specific genetic background (the underlying mutation being *lgl*^*4*^). In the fly, just as in humans, one would expect tumors with the same genotype to behave differently in different tissues, and additionally expect different combinations of mutations to result in different phenotypes. Despite this, work carried out in the human breast cancer cell line MCF7 shows that the majority of hits tested give the same phenotypes and thereby will have relevance to human disease. This is most clearly seen when testing cohesin subunits in the fly and in MCF7 cells: STAG1 and STAG2 both promote invasion when their expression is knocked down, whereas other cohesin subunits do not, recapitulating the effect seen within the fly screen.

### Resource Availability

#### Lead Contact

Further information and requests for resources and reagents should be directed to and will be fulfilled by the lead contact, Marios Georgiou (marios.georgiou@nottingham.ac.uk).

#### Materials Availability

All unique/stable reagents generated in this study will be made available on request, but we may require a payment and/or a completed Materials Transfer Agreement if there is potential for commercial application.

#### Data and Code Availability

The accession number for the microarray data reported in this paper is GEO: GSE137773. An online searchable database with all results from the screen, including raw high-resolution images for each RNAi line, is available at https://flycancerscreen.nottingham.ac.uk.

## Methods

All methods can be found in the accompanying Transparent Methods supplemental file.

## Supplemental Information

Document S1. Transparent Methods and Figures S1–S8

Table S1. Full Database, Related to Figures 2 and 3

Table S2. Level of Similarity between Two RNAi Lines Targeting the Same Gene, Related to Figure 3 See also Figure S2.

Table S3. Hits For all Categories, Related to Figure 3

Table S4. Lists of Genes within Clusters and Associated GO Terms See also Figure 3

Table S5. Genes Showing a Significant Change in Expression Following STAG2KD in MCF7 Cells, Related to Figure 6 See also Figure S6.

Document S2. Data S1 and S2 Data S1. Cytoscape network file for interaction map of invasive genes, Related to Figure 4. Data S2. Cytoscape network file for interaction map of genes misregulated by STAG2KD that affect cell-cell junctions, Related to Figure 6 See also Figure S6.
