# Supplementary material for: A Genetic Analysis of Tumor Progression in Drosophila Identifies the Cohesin Complex as a Suppressor of Individual and Collective Cell Invasion
Source: iScience. 2020 Jun 4;23(6):101237. doi: 10.1016/j.isci.2020.101237 (PMC7317029; doi:10.1016/j.isci.2020.101237)
Supplement: Document S1. Transparent Methods and Figures S1–S8 [file mmc1.pdf]

iScience, Volume *23*

# Supplemental Information

## A Genetic Analysis of Tumor Progression in *Drosophila* Identifies the Cohesin Complex as a Suppressor of Individual and Collective Cell Invasion

**Brenda Canales Coutiño, Zoe E. Cornhill, Africa Couto, Natalie A. Mack, Alexandra D. Rusu, Usha Nagarajan, Yuen Ngan Fan, Marina R. Hadjicharalambous, Marcos Castellanos Uribe, Amy Burrows, Anbarasu Lourdusamy, Ruman Rahman, Sean T. May, and Marios Georgiou**

# Figure S1

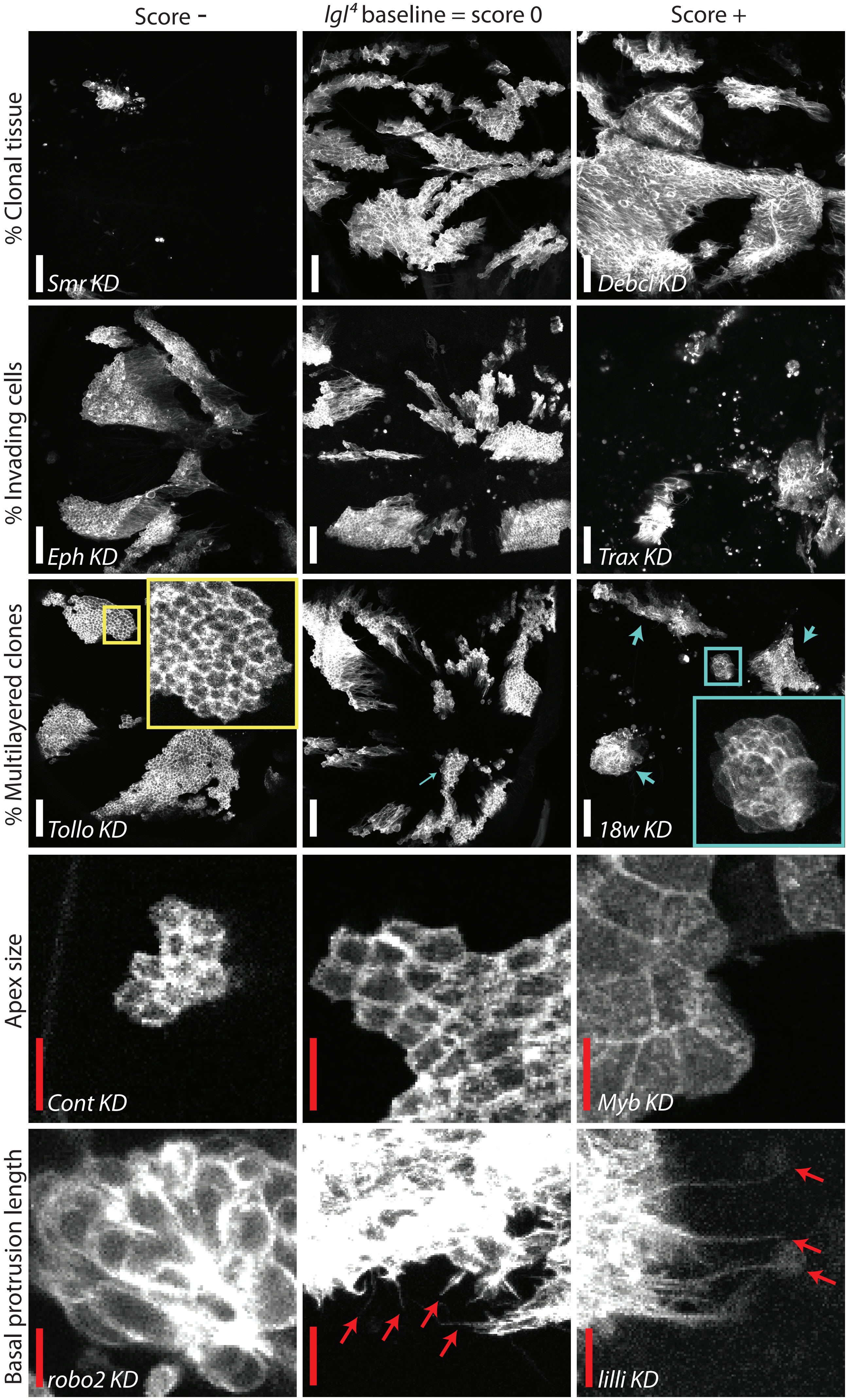

Score -
lgl4 baseline = score 0
Score +
% Clonal tissue
% Invading cells
% Multilayered clones
Apex size
Basal protrusion length
Smr KD
Debcl KD
Eph KD
Trax KD
Tollo KD
18w KD
Cont KD
Myb KD
robo2 KD
lilli KD

**Figure S1: Semi-quantitative scoring system employed to systematically record phenotypes across phenotypic categories.** Related to Table S1 and Figures 2-3.
Rows show five example categories from the 33 categories within the database to illustrate our scoring method: percentage of clonal tissue within the fly pupal notum (i.e. GFP-positive area versus GFP-negative area); percentage of invading cells against total GFP-positive tissue; percentage of multilayered clones against total GFP-positive tissue; average apex size of epithelial cells; average length of basal protrusions. Columns show scores given: central column shows a score of 0 for the *lgl*$^{4}$ mutant background; right column shows positive scores of +1 or +2, which indicate an increase in phenotype compared to the *lgl*$^{4}$ baseline; left column shows negative scores of -1 or -2, which indicate a decrease in phenotype compared to *lgl*$^{4}$ baseline. Panel names on right and left columns refer to the specific gene knocked down in an *lgl*$^{4}$ mutant background. Yellow box indicates a magnified monolayered clone. Turquoise box indicates a magnified multilayered clone; turquoise arrows highlight multilayered clones within the notum (others are monolayered). Red arrows point to basal protrusions. White scale bar: 10μm; red scale bar: 5μm.

**Figure S2**

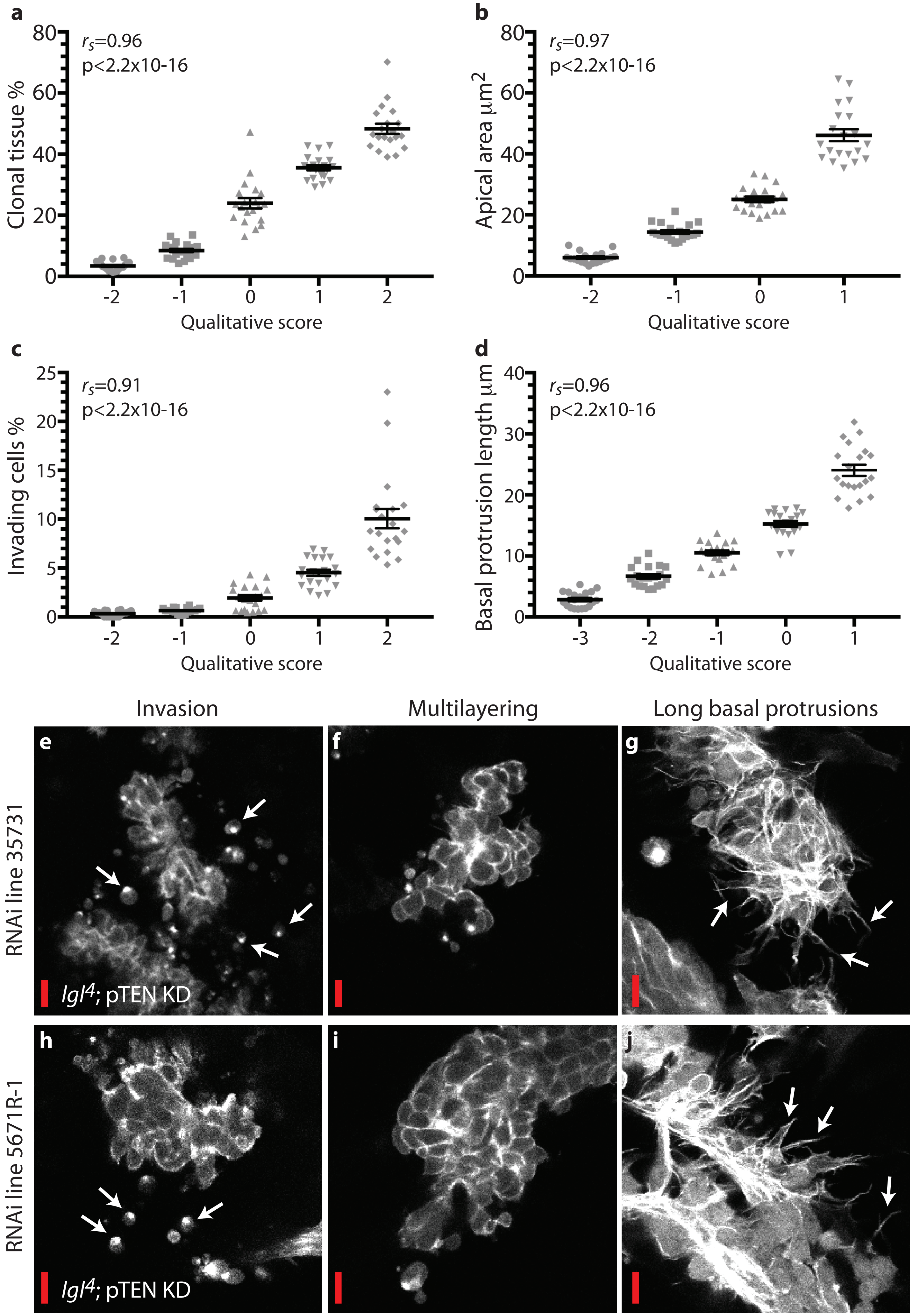
a
$r_S$=0.96
p<2.2x10-16
Clonal tissue %
Qualitative score
b
$r_S$=0.97
p<2.2x10-16
Apical area µm2
Qualitative score
c
$r_S$=0.91
p<2.2x10-16
Invading cells %
Qualitative score
d
$r_S$=0.96
p<2.2x10-16
Basal protrusion length µm
Qualitative score
Invasion
Multilayering
Long basal protrusions
RNAi line 35731
RNAi line 5671R-1
e
f
g
h
i
j
lgl4; pTEN KD
lgl4; pTEN KD

**Figure S2: Quality control.** Related to Table S2 and Figure 3.
**(a-d)** Graphs comparing qualitative scores given to individual animals against the quantitative measure for a range of phenotypes, including: (a) percentage clonal tissue; (b) apical area; (c) percentage invading cells (d) basal protrusion length (n= 20 animals for each category of qualitative score). (e-j) A comparison of the phenotypes observed when two independent RNAi lines (35731-GD and 5671R-1) were used to target the same gene (pTEN). Both showed strong effects on invasion (e and h), multilayering (f and i), and basal protrusion length (g and j). Arrows highlight: invading cells (f and i); long basal protrusions (g and j). Scale bars = 10 μm. Error bars represent ± s.e.m.

**Figure S3**

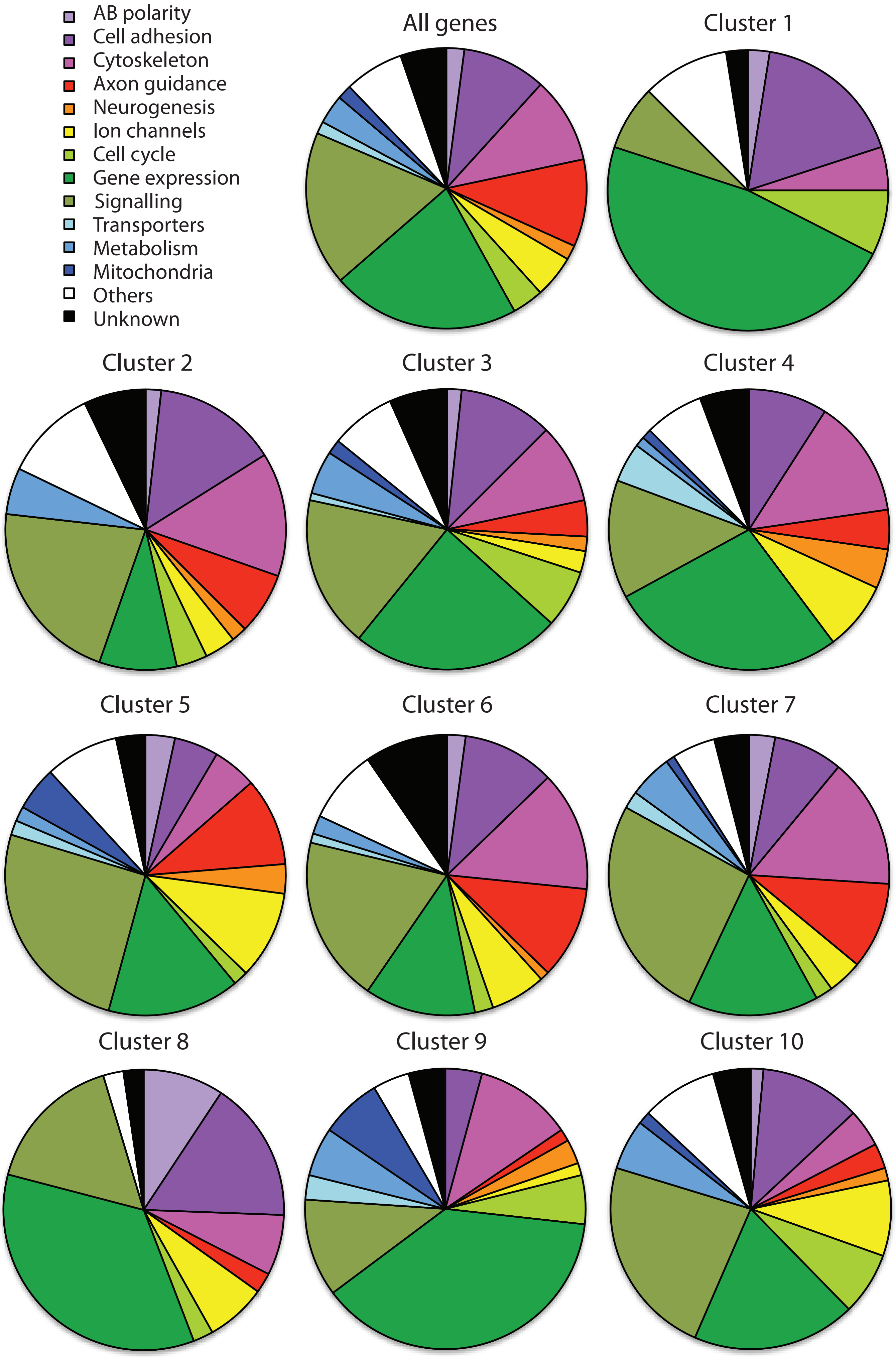
AB polarity
Cell adhesion
Cytoskeleton
Axon guidance
Neurogenesis
Ion channels
Cell cycle
Gene expression
Signalling
Transporters
Metabolism
Mitochondria
Others
Unknown
All genes
Cluster 1
Cluster 2
Cluster 3
Cluster 4
Cluster 5
Cluster 6
Cluster 7
Cluster 8
Cluster 9
Cluster 10

**Figure S3: An analysis of gene function for genes within phenotypic clusters.** Related to Figure 3. Pie charts illustrating the range of biological functions for all 497 genes included in the screen (all genes), as well as those functions that were enriched within phenotypic clusters (clusters 1-10).

# Figure S4

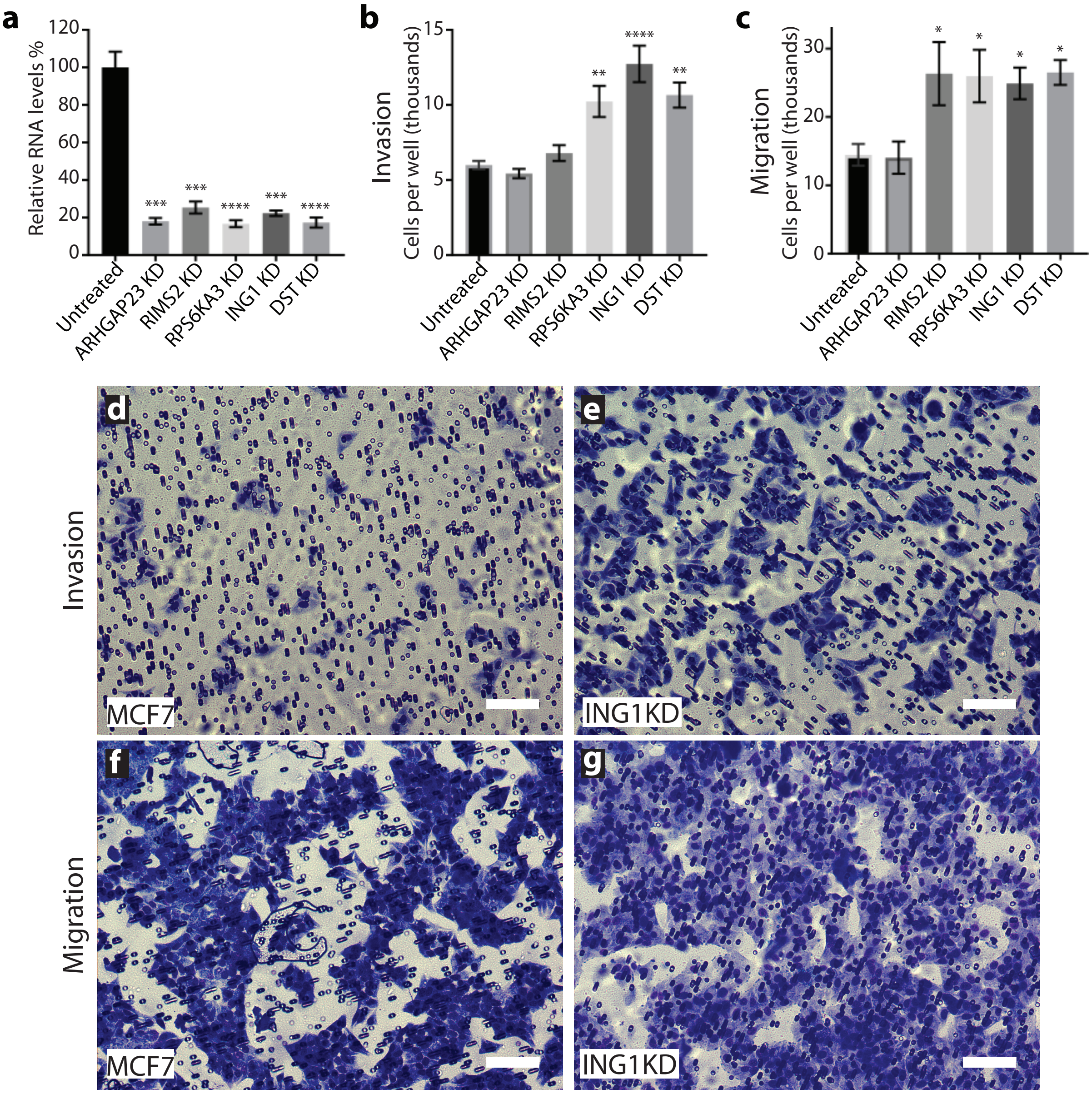
a
Relative RNA levels %
120
100
80
60
40
20
0
***
***
****
***
****
Untreated
ARHGAP23 KD
RIMS2 KD
RPS6KA3 KD
ING1 KD
DST KD
b
Invasion
Cells per well (thousands)
15
10
5
0
**
****
**
Untreated
ARHGAP23 KD
RIMS2 KD
RPS6KA3 KD
ING1 KD
DST KD
c
Migration
Cells per well (thousands)
30
20
10
0
*
*
*
*
Untreated
ARHGAP23 KD
RIMS2 KD
RPS6KA3 KD
ING1 KD
DST KD
Invasion
Migration
d
MCF7
e
ING1KD
f
MCF7
g
ING1KD

**Figure S4: In vitro transwell migration and invasion assays for human orthologues of a selection of highly invasive fly genotypes.** Related to Figure 5.
**(a-c)** Quantification of invasion (b) and migration (c), showing the number of MCF7 cells (a human breast adenocarcinoma cell line), untreated or transfected with siRNA targeting the indicated gene, which migrated through an 8μm pore membrane. For the invasion assay the membrane was coated with a layer of Matrigel (n=3 for each assay). Relative mRNA levels, determined by qRT-PCR, are shown in (a). Error bars represent ± s.e.m. One-way ANOVA with Dunnett's post hoc test for multiple comparisons was performed to determine statistical significance. **(d-g)** Representative images from the invasion and migration assays, showing untreated MCF7 cells and ING1KD cells. Staining intensity (purple) is directly proportional to the number of cells that have invaded through the ECM (d-e) or migrated through the 8μm pore membrane (f-g). Scale bar: 100μm.

# Figure S5

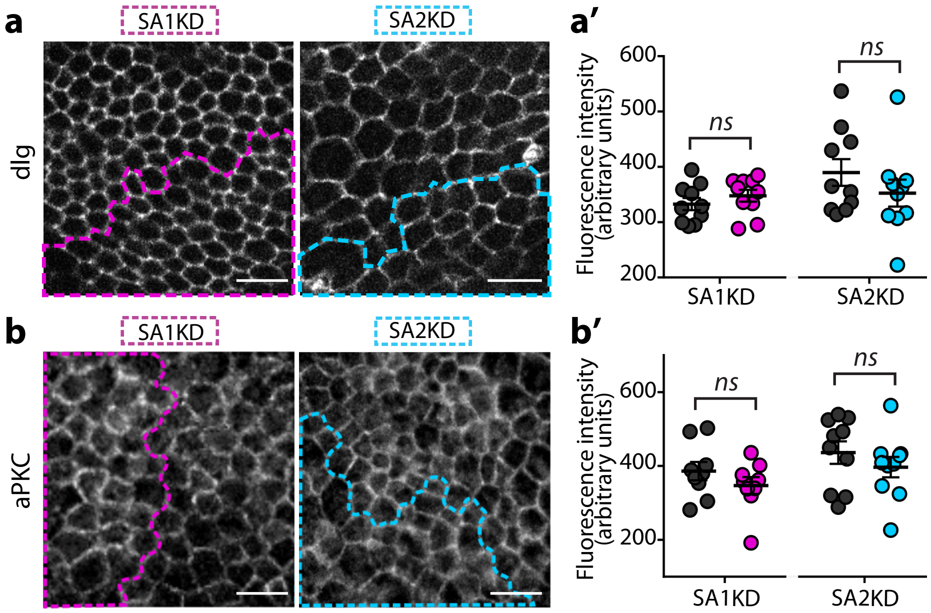

a
SA1KD
SA2KD
dlg
a'
Fluorescence intensity (arbitrary units)
600
500
400
300
200
ns
ns
SA1KD
SA2KD
b
SA1KD
SA2KD
aPKC
b'
Fluorescence intensity (arbitrary units)
600
400
200
ns
ns
SA1KD
SA2KD

**Figure S5: SA1 or SA2KD does not affect Dlg or aPKC localisation.** Related to Figure 6.
**(a-b)** SA1 or SA2KD clones, highlighted by magenta and cyan dashed lines, respectively, show normal dlg (a) and aPKC (b) localisation. **(a'-b')** Quantification shows fluorescence intensity at the level of highest intensity (n=100 junctions from 10 animals for each genotype). Scale bars: 10µm. Error bars = ± s.e.m. Statistical analysis: Student's T test.

# Figure S6

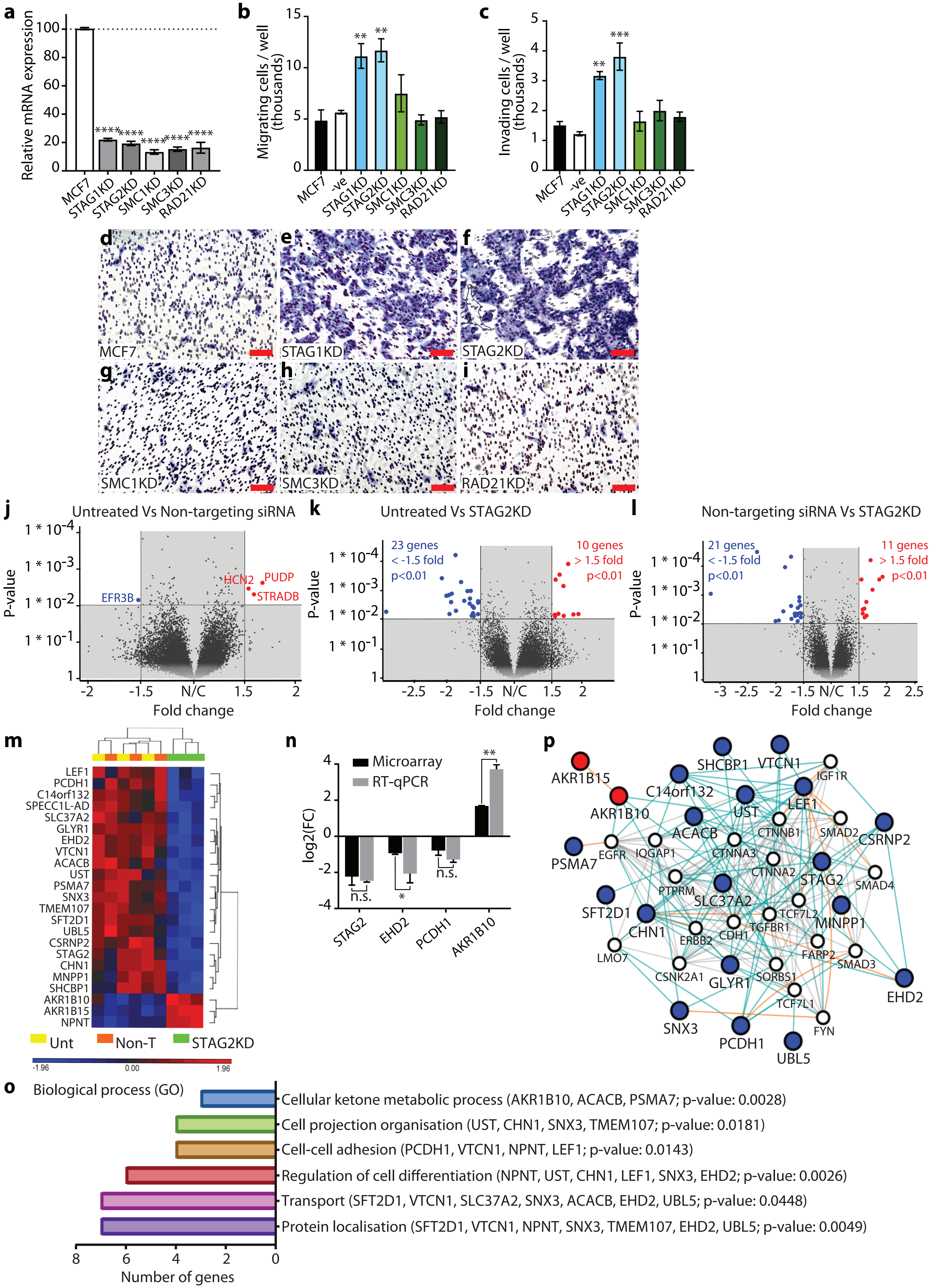
a
Relative mRNA expression
MCF7
STAG1KD
STAG2KD
SMC1KD
SMC3KD
RAD21KD
b
Migrating cells / well (thousands)
-ve
c
Invading cells / well (thousands)
d
MCF7
e
STAG1KD
f
STAG2KD
g
SMC1KD
h
SMC3KD
i
RAD21KD
j
Untreated Vs Non-targeting siRNA
EFR3B
HCN2
PUDP
STRADB
P-value
Fold change
k
Untreated Vs STAG2KD
23 genes
< -1.5 fold
p<0.01
10 genes
> 1.5 fold
p<0.01
l
Non-targeting siRNA Vs STAG2KD
21 genes
< -1.5 fold
p<0.01
11 genes
> 1.5 fold
p<0.01
m
LEF1
PCDH1
C14orf132
SPECC1L-AD
SLC37A2
GLYR1
EHD2
VTCN1
ACACB
UST
PSMA7
SNX3
TMEM107
SFT2D1
UBL5
CSRNP2
STAG2
CHN1
MNPP1
SHCBP1
AKR1B10
AKR1B15
NPNT
Unt
Non-T
STAG2KD
-1.96
0.00
1.96
n
Microarray
RT-qPCR
log2(FC)
n.s.
STAG2
EHD2
PCDH1
AKR1B10
p
AKR1B15
AKR1B10
SHCBP1
VTCN1
C14orf132
IGF1R
LEF1
UST
ACACB
CTNNB1
SMAD2
CSRNP2
PSMA7
EGFR
IQGAP1
CTNNA3
CTNNA2
STAG2
SMAD4
PTPRM
SLC37A2
SFT2D1
TCF7L2
MINPP1
CHN1
CDH1
TGFBR1
ERBB2
LMO7
FARP2
SMAD3
CSNK2A1
GLYR1
SORBS1
EHD2
TCF7L1
SNX3
PCDH1
FYN
UBL5
o
Biological process (GO)
Cellular ketone metabolic process (AKR1B10, ACACB, PSMA7; p-value: 0.0028)
Cell projection organisation (UST, CHN1, SNX3, TMEM107; p-value: 0.0181)
Cell-cell adhesion (PCDH1, VTCN1, NPNT, LEF1; p-value: 0.0143)
Regulation of cell differentiation (NPNT, UST, CHN1, LEF1, SNX3, EHD2; p-value: 0.0026)
Transport (SFT2D1, VTCN1, SLC37A2, SNX3, ACACB, EHD2, UBL5; p-value: 0.0448)
Protein localisation (SFT2D1, VTCN1, NPNT, SNX3, TMEM107, EHD2, UBL5; p-value: 0.0049)
Number of genes

**Figure S6: STAG1 and STAG2 are invasion suppressors in human cells.** Related to Figure 6 and Table S5.

**(a-i)** In vitro transwell migration and invasion assays with the human breast adenocarcinoma cell line, MCF7, with or without the knockdown of human somatic cohesin complex subunits. **(a)** Relative mRNA levels following KD, determined by qRT-PCR. **(b-c)** Quantification of migration (b) and invasion (c) showing the number of MCF7 cells, untreated or transfected with siRNA targeting the indicated gene, which migrated through an 8μm pore membrane. For the invasion assay, the membrane was coated with a layer of Matrigel. n=3 for each assay. **(d-i)** Representative images of the invasion assay, showing dramatically increased invasion specifically when STAG subunits are knocked down. Staining intensity (purple) is directly proportional to the number of cells that have invaded through the ECM. Scale bar=100μm. **(j-l)** Volcano plot of significance level versus fold change in genetic expression from treated and untreated MCF7 cells. Each grey dot represents a gene that had no significant change in expression i.e. that had a p value greater than 0.01 and an expression fold change between -1.5 and 1.5. Each blue and red dot represents a gene that was downregulated or upregulated, respectively, compared to the control. Differentially expressed genes between STAG2KD cells and untreated MCF7 cells (unt) or MCF7 cells treated with non-targeting siRNA (nonT) are shown in (k) and (l) respectively; (j) is the negative control. To reduce the possibility of false positives, only genes that were differentially expressed in both STAG2KD against unt and nonT were selected. **(m)** Heat-map representation of unsupervised clustering of the 23 differentially expressed genes by STAG2KD in MCF7 cells. Each column represents a sample. Yellow: unt; orange: nonT; green: STAG2 siRNA treated. Colour code represents log2 of the fold change of expression: blue, downregulated; red, upregulated. Horizontal and vertical clusters were created based in Euclidean distance. **(n)** Bar chart comparing microarray and RT-qPCR for four genes with differential expression. **(o)** Gene Ontology (GO) terms reaching Bonferroni corrected significance for enrichment amongst genes that had significantly altered their expression by STAG2KD. **(p)** Interaction network of differentially expressed genes by STAG2KD and adherens junction KEGG pathway proteins. The GeneMANIA plug-in for Cytoscape was used to generate an interaction network based on previously documented interactions. White nodes mark AJ components; red nodes: genes upregulated by STAG2KD; blue nodes: genes downregulated by STAG2KD. Orange lines: physical interactions; cyan lines: genetic interactions; grey lines: interactions between AJ genes. Error bars represent ± s.e.m. Student's T test or One-way ANOVA with Dunnett's post hoc test for multiple comparisons was performed to determine statistical significance.

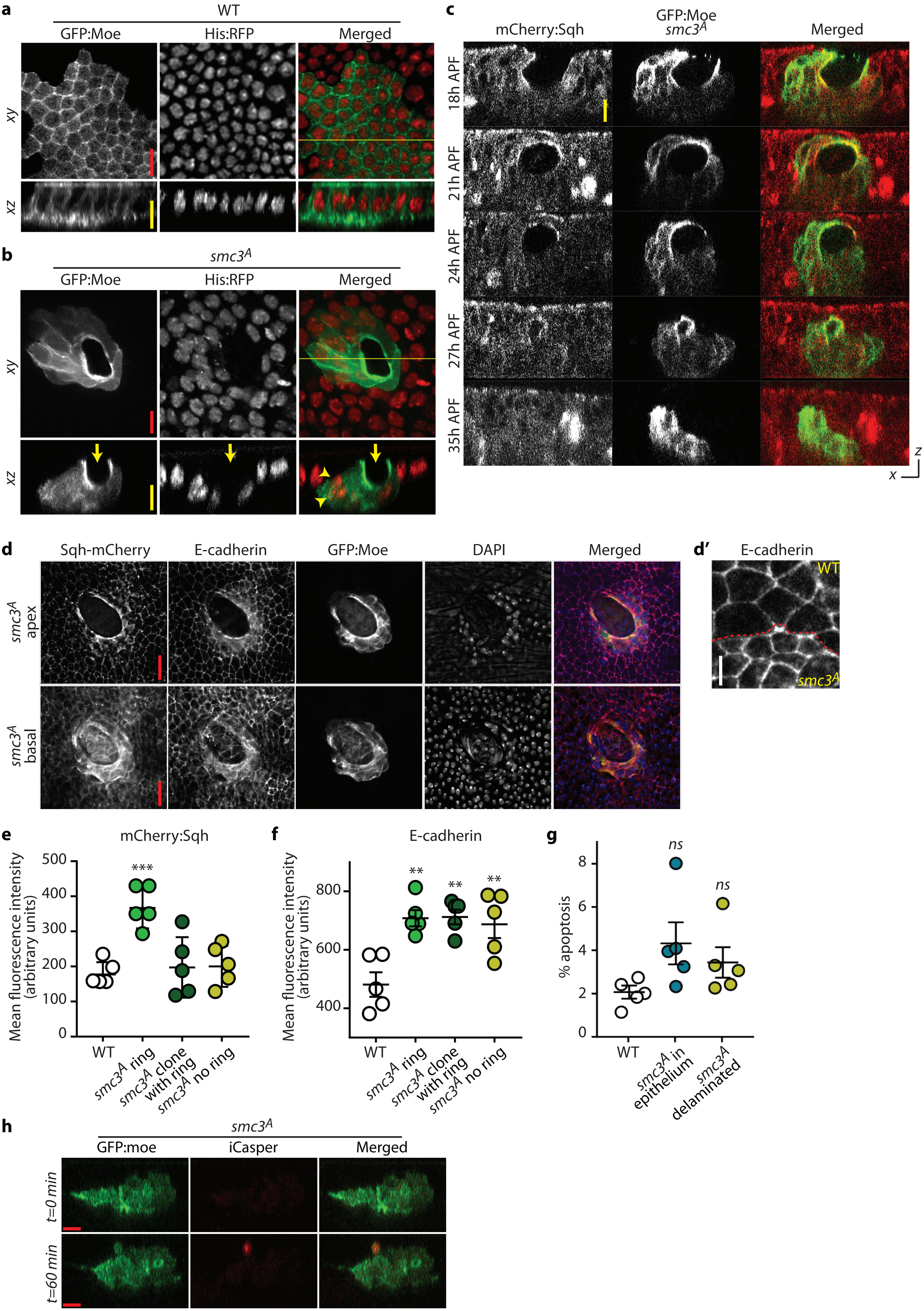

Figure S7
a
WT
GFP:Moe
His:RFP
Merged
xy
xz
b
smc3A
GFP:Moe
His:RFP
Merged
xy
xz
c
mCherry:Sqh
GFP:Moe smc3A
Merged
18h APF
21h APF
24h APF
27h APF
35h APF
z
x
d
Sqh-mCherry
E-cadherin
GFP:Moe
DAPI
Merged
smc3A apex
smc3A basal
d'
E-cadherin
WT
smc3A
e
mCherry:Sqh
Mean fluorescence intensity (arbitrary units)
500
400
300
200
100
0
***
WT
smc3A ring
smc3A clone with ring
smc3A no ring
f
E-cadherin
Mean fluorescence intensity (arbitrary units)
800
600
400
**
**
**
WT
smc3A ring
smc3A clone with ring
smc3A no ring
g
% apoptosis
8
6
4
2
0
ns
ns
WT
smc3A in epithelium
smc3A delaminated
h
smc3A
GFP:moe
iCasper
Merged
t=0 min
t=60 min

**Figure S7: *smc3* LOF induces a supracellular actomyosin ring, which leads to invagination and clonal extrusion.** Related to Figure 7.

**(a-b)** GFP:moe positively marked clones (green) and ubiquitously expressed Histone:RFP (red). Wild type tissue organisation is uniform; the orthogonal (*xz*) view shows a monolayer of cells (a). *smc3*$^{A}$ clones frequently contain a supracellular actin-rich ring (b). No Histone:RFP signal is observed inside the ring. The *xz* view shows that the actin ring induces invagination (arrows); the nuclei of *smc3*$^{A}$ cells are displaced basally (arrowheads). Yellow line shows position of *xz* slice shown. **(c)** Lateral view of a GFP:moe positively marked *smc3*$^{A}$ clone (green) with additional mCherry:Sqh labelling (red). Animal was imaged live for approximately 15 hours. At 18h APF, the actin ring induces tissue invagination. At 21h APF, the actin ring closes, forming a central lumen. The mutant clone is extruded from the epithelium, and the actin ring contracts over time, closing the internal lumen (35h APF). **(d)** A dissected *Drosophila* notum containing a GFP:moe positively marked *smc3*$^{A}$ clone with additional mCherry:Sqh labelling. The tissue is also stained for E-cadherin and DAPI. An apical confocal section shows no protein localisation or DAPI nuclear stain within the actin-rich ring. A more basal confocal section shows E-cadherin and DAPI staining, marking the adherens junctions and nuclear material of invaginated cells. **(d')** A higher magnification image of the E-cadherin stain, comparing *smc3*$^{A}$ tissue with neighbouring WT tissue. **(e-f)** Quantification of mean fluorescence intensity of mCherry:Sqh (e) and E-Cadherin (f) in WT clones and *smc3*$^{A}$ clones, with or without actin-rich rings. If a ring was present, measurements were taken at the ring, or at junctions outside the ring. 50 measurements from 5 animals were quantified, each dot represents one animal. Note the increased Sqh levels specifically at the ring, whereas cortical E-cadherin is significantly increased throughout the mutant clone. **(g)** Quantification of iCasper positive cells within WT clones, *smc3*$^{A}$ clones that are still connected to the epithelial sheet, and extruded *smc3*$^{A}$ clones. n=5 animals, all the labelled cells within each animal were quantified, each dot represents one animal. **(h)** Confocal images of a delaminated iCasper (red) and GFP:Moe (green) labelled *smc3*$^{A}$ clone showing minimal apoptosis over 1h post extrusion. Red scale bars: 10μm; white scale bar: 5μm; yellow scale bars: 10μm in the *xz* plane. Error bars = ± s.e.m. Statistical analysis: Student's T test.

**Figure S8**

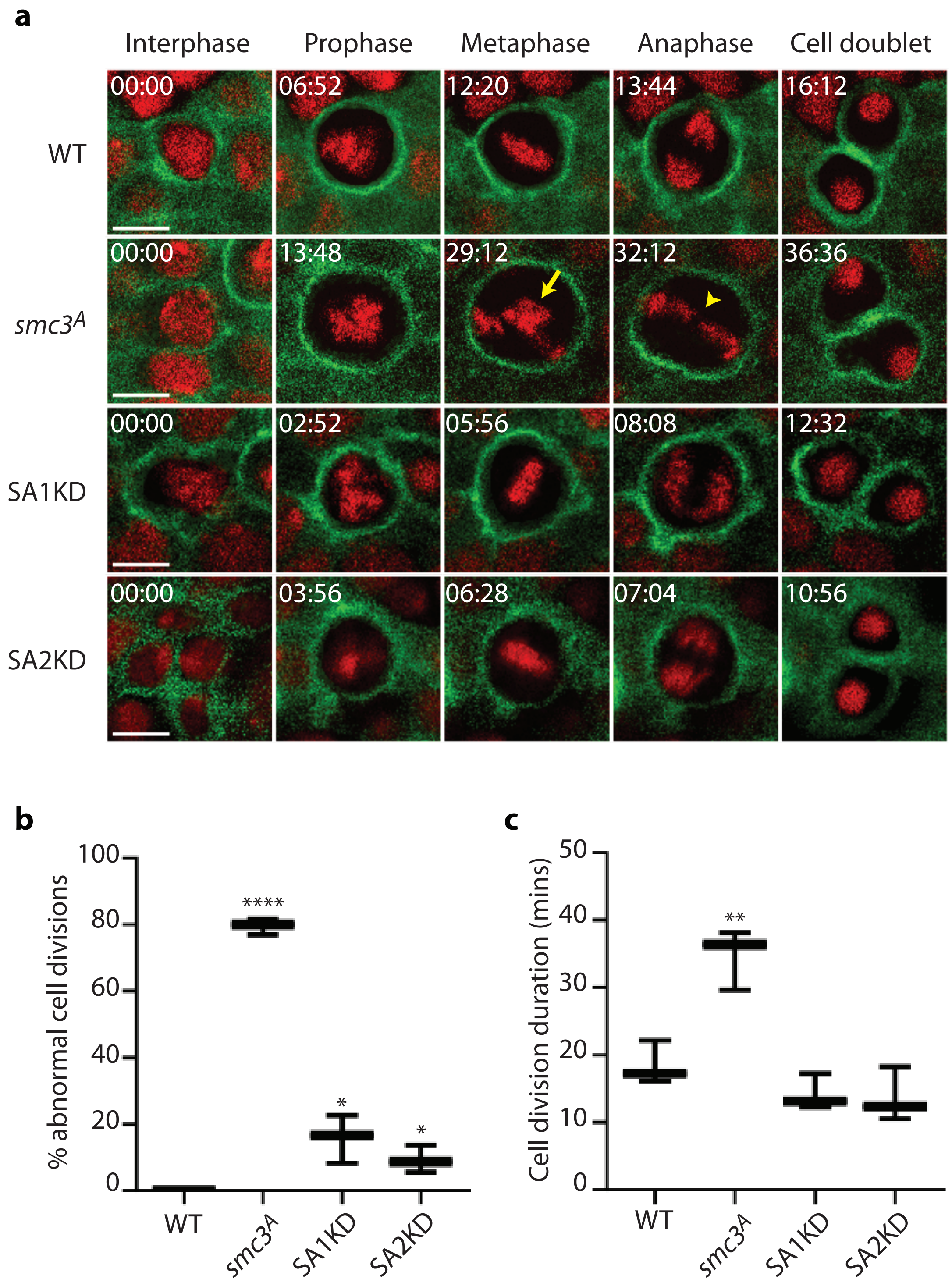

a
Interphase
Prophase
Metaphase
Anaphase
Cell doublet
WT
00:00
06:52
12:20
13:44
16:12
smc3A
00:00
13:48
29:12
32:12
36:36
SA1KD
00:00
02:52
05:56
08:08
12:32
SA2KD
00:00
03:56
06:28
07:04
10:56
b
% abnormal cell divisions
100
80
60
40
20
0
****
*
*
WT
smc3A
SA1KD
SA2KD
c
Cell division duration (mins)
50
40
30
20
10
0
**
WT
smc3A
SA1KD
SA2KD

**Figure S8: Live imaging shows frequent defective chromosome dynamics in *smc3*$^{A}$ clones during cell division.** Related to Movies S3-S6 and Figures 6-7.
**(a)** In vivo imaging of WT, *smc3*$^{A}$, SA1KD, and SA2KD clones, labelled with GFP:Moe and Histone:RFP, allowing live cell analysis of chromosomal dynamics during cell division. Scale bar: 5μm. Time stamp in top left corner of each panel. Arrow highlights defective chromosome alignment; arrowhead defective chromosome separation. **(b-c)** Quantification of percentage abnormal cell divisions (b) and cell division duration (c). n=40 cells from 4 animals for each genotype. Error bars = ± s.e.m. Statistical analysis: Student's T test.

Transparent Methods

## Transgenic *Drosophila* stocks and crosses

Flystocks and crosses were raised on standard cornmeal and yeast medium at 25$^{O}$C.
The following stocks were used: Ubx-FLP (#42718), *l(2)gl*$^{4}$ neoFRT40A /CyO (#36289), tubP-GAL80 neoFRT40A (#5192), Pnr-GAL4 (#25758), UAS-GMA (#31776), tubP-GAL80 neoFRT19A (#5132), UAS-p35 (#6298), sna[Sco]/CyO; PBac{y[+mDint2] w[+mC]=UAS-iCasper-noGFP-T2A-HO1}VK00005/TM6B (#64186), sqh-mCherry (#59024) were obtained from Bloomington *Drosophila* Stock Center (Indiana, USA).
UAS-RNAi stocks were obtained from VDRC Stock Center (Vienna, Austria) and NIG (Japan).
w$^{1118}$; P{GD12222} v27988 and w$^{1118}$; P{GD12222}v27989 were obtained from the *Drosophila* Genetic Resource Center (DGRC).
Ubx-FLP; FRT40A/Cyo-GFP, Pnr-GAL4, UAS-GFP:Moe/TM6b,
Ubx-FLP; *l(2)gl*$^{4}$, neoFRT40A /Cyo-GFP; Pnr-GAL4, UAS-GFP:Moe/TM6b,
Ubi-His:RFP/CyO, and
w; tub-Gal80, FRT40A; MKRS/TM6b are lab stocks.
The following strains were recombined in the lab to create new strains: Pnr-GAL4 and UAS-GFP:Moe (chromosome III), Ubx-FLP and *Dlg*$^{52}$ (chromosome X), Pnr-GAL4 and neoFRT82b *scrib*$^{1}$ (chromosome III).
We designed an F2 RNAi screen as follows: tubP-GAL80 FRT40A; MKRS/TM6b Tb Hu virgin flies were crossed to UAS-RNAi lines on chromosome III; male and humoral F1 progeny of this cross of genotype tubP-GAL80 FRT40A /+; UAS-RNAi/TM6b Tb Hu were crossed to Ubx-FLP; *lgl*$^{4}$ FRT40A /CyO, Kr-GFP; Pnr-GAL4, UAS-GFP:Moe/TM6b Tb Hu virgin females; male and female F2 progeny of genotype Ubx-FLP/+; *lgl*$^{4}$ FRT40A / tubP-GAL80 FRT40A; Pnr-GAL4, UAS-GFP:Moe/UAS-RNAi were mounted for confocal imaging.

## Dissections and live imaging

White pupae of the appropriate genotype were selected and aged at 29$^{O}$C for 18 to 22 hours. Nota from aged pupae were dissected in PBS and the tissue fixed in 4% formaldehyde for 20 min at room temperature, before being blocked and permeabilised with PBS containing 0.2% BSA, 5% NGS, 0.1% Triton X-100.
For live imaging, aged animals with the appropriate genotype were prepared by cutting a window in the pupal case, attached to a slide with double-sided sticky tape. A coverslip with a drop of injection oil was then placed over the notum, supported by coverslips at either end to allow imaging on inverted confocal microscopes. Live imaging was performed with either a Leica SP2 inverted confocal microscope equipped with a × 40/1.25 NA oil objective with PL APO correction, a Zeiss LSM880 inverted confocal microscope equipped with a x 40/ 1.30 NA oil Ph3 M27 objective or a Zeiss LSM5 Exciter AxioObserver equipped with an EC Plan-NeoFluar × 40/1.30 oil lens. Z-series were acquired using 1 µm z-sectioning. For time-lapse experiments z-series were acquired every 3 minutes for 2 hours.
Images from fixed samples were acquired on a Zeiss LSM880 inverted confocal microscope using a x 40/ 1.30 NA oil Ph3 M27 objective. Z-series were acquired using 0.5 µm z-sectioning.

## Antibodies

We used the following primary antibodies at the indicated dilutions for this study: rat anti-E-Cad [1:100, DSHB (DCAD2)], mouse anti-Armadillo (1:100, DSHB), rat anti- **α**-Catenin [1:100, DSHB (DCAT1)], mouse anti-Fasciclin III [1:400, DSHB (7G10)], mouse anti-Discs Large [1:100, DSHB (4F3)], rabbit anti-PKC zeta [1:50, Santa Cruz (sc-216-G)], rabbit anti-pMad [1:250, Abcam (ab52903)]. Secondary antibodies from Molecular Probes were Alexa Fluor 488, 546 and 633.

## Cell Culture

MCF-7 human breast carcinoma cells were obtained from Dr Anna Grabowska (Faculty of Medicine & Health Sciences, University of Nottingham). Cells were cultured in RPMI medium without phenol red (Invitrogen) supplemented with 10% fetal bovine serum (Sigma-Aldrich) and 20mM L-glutamine (Sigma) and grown in T75 culture flasks at 37°C in a 5% CO2 atmosphere.

To knockdown gene expression, MCF-7 cells were incubated for 48 hours with transfection media containing DharmaFect1 (Dharmacon) transfection reagent and ON-TARGET plus SMARTpool siRNA (Dharmacon) against ArhGAP23, DST, RPS6KA3, RIMS2, ING1, STAG1, STAG2, SMC1, SMC3 and RAD21. ON-TARGETplus Non-targeting siRNA (Dharmacon) was used as a negative control and siGLO controls (Dharmacon) for transfection optimisation experiments.

After the transfection, cells with and without the knockdown were harvested and plated at appropriate density in hanging inserts with 8μm pore (Millipore). For invasion experiments, the inserts were covered with 75μg extracellular matrix (Corning). Cells were plated in 1% FBS RPMI and 10% FBS RPMI was used underneath the inserts. The amount of migrating or invading cells was assessed after a 48 hours incubation. Migrating or invading cells were detached from the bottom of the inserts using accumax cell detachment solution (Merk Millipore). The fluorescence intensity of the cell suspension was measured using 480/520 nm filters and optimal gain on a BMG fluorescent plate reader after a 15 minutes incubation with CyQuant GR dye (Thermo Fisher Scientific). Images of the migrating or invading cells were acquired using a 10x/ 0.3 NA objective on a Zeiss Axioplan/Axiophot brightfield microscope after inserts were stained using a Shandon Kwik-Diff stain kit (thermo fisher scientific) following the manufacturer's instructions. A background correction was performed using Gimp Software.

Total cellular RNA was extracted from un-transfected cells, cells featuring knockdown and cells transfected with ON-TARGETplus Non-targeting Control siRNAs (Dharmacon) using TRIzol (Thermo Fisher Scientific) following the manufacturer's instructions. The concentration and purity of RNA were determined by spectrophotometry. cDNA was synthetized using RevertAid First Strand cDNA Synthesis Kit [Thermo Fisher Scientific (k1621)] following the manufacturer's instructions. Quantitative real-time PCR (RT-PCR) was performed with A C1000 thermal cycler CFX96 RT system. Non-universal primers were designed using primer 3. Samples were labelled using IQ SYBR Green qPCR Master Mix (BioRad). Primer specificity and efficiency was confirmed by standard and melting curve analyses. Relative transcript levels were calculated relative to the transcript level of the reference gene GAPDH using the fold change method:

$$\Delta \text{Cq} = \text{mean Cq (target gene)} - \text{mean Cq (housekeeping gene)}$$

$$\text{E}\Delta\text{Cq} = \frac{\text{power (PCR efficiency for the housekeeping gene,mean Cq (target gene))}}{\text{power (PCR efficiency for the target gene,mean Cq (housekeeping gene)}}, \text{FC} \frac{\text{E}\Delta\text{Cq}}{\text{mean E}\Delta\text{Cq}}.$$

## Primer list

| Primer name | Sequence 5' | Sequence 3' |
|---|---|---|
| GAPDH human | ATGTTCGTCATGGGTGTGAA | GTCTTCTGGGTGGCAGTGAT |
| ING1 human | TACTGTCTGTGCAACCAGGT | TTCTCCAGGGCTTTGTCCAT |
| ARHGAP23 human | CCGATGAATGGAGTCGCCTT | ATGAAGTGGCGCAGAGTGAA |
| DST human | TACTGCCCTGGTCACTCTCAT | TACATCTGCTACCGGCCAC |
| RPS6KA3 human | ACCACATCTAGTAAAGGGTGCC | TTCACAGGGCTGTTGAGGTG |
| RIMS2 human | ACAGCAAGAACAGAAGGGTGA | CACATAACCTTGTTTGAGCGT |
| STAG1 | GGCCAGCCGAAGTTAGAAGA | CCATTAGACCCCGAACAGCA |
| STAG2 | AGACATGCCTGAGCAGATTGT | AAACGCAGCAAGTCCTCCTT |
| SMC1 | ATCCACAGCTACAAGCCAGC | TTGCAAGTCGACTGCTCCTT |
| SMC3 | TGGAGTTGGAATTAGGGTGTCA | GTCCACCTGAAAGCTGTTGC |

| | | |
|---|---|---|
| RAD21 | TGTATCAATGGGTGGGCCTG | GGCTCCAATGCAAATGCTTCT |
| PCDH1 | GTCATCCAGGTGAAGGCCAA | CGGTCCTTAGCAAGCACTGA |
| EHD2 | GGTGCGAGTTCACGCTTACA | AGATGACGGGCAGTTTGAGG |
| AKR1B10 | CAGCAACAGAGAGCAGGACG | TGCCAAGAGGAGACTTCCAA |

### Interaction map

The interaction network was generated by integrating publicly available data to obtain a unified map of genes involved in cancer invasion. The data resources used were DroID (www.droidb.org, version 2018_08), Flybase (version FB2018_03) and BioGRID (version 3.5.165). PubMed ID was used as a unique identifier for each interaction, to prevent the inclusion of redundant information. Interactions were included into further consideration only if both interactors were hits in the categories of 'invasion', 'multilayering', 'cell body rounding', 'lethals' or if the interactor was not part of the screen yet linked an otherwise not interacting gene from any of these categories. The resulting network was drawn using Cytoscape 3.6 (http://www.cytoscape.org). MCODE v1.5.1 algorithm was used to identify subnetwork complexes of highly interconnected nodes.

### Transcriptomics

Whole-genome transcriptome analysis of transfected and un-transfected (both un-treated and treated with non-targeting siRNA) MCF-7 cells was conducted at the Nottingham Arabidopsis Stock Centre (NASC). The RNA concentration and quality was assessed using the Agilent 2100 Bioanalyzer (Agilent Technologies Inc., Palo Alto, CA) and the RNA 600 Nano Kit (Caliper Life Sciences, Mountain View, CA). Samples with a minimum RNA concentration of 100 ng/µl and RNA Integrity Number (RIN) ≥ 8 were used for gene expression analysis. Single stranded complimentary DNA was prepared from 200 ng of total RNA as per the GeneChip™ WT PLUS Reagent Kit (Applied Biosystems and Affymetrix). Total RNA was first converted to cDNA, followed by *in vivo* transcription to make cRNA. Single stranded cDNA was synthesised, end labelled and hybridised for 16 h at 45℃ to Clariom™ S Assay arrays (Thermo Fisher Scientific).
Gene expression data were analysed using Partek Genomics Suite 6.6 software (Partek Incorporated). The raw CEL files were normalised using the RMA background correction with quantile normalisation, log base 2 transformation and mean probe-set summarisation with adjustment for GC content. Differentially expressed genes (DEG) were identified by a two-way ANOVA. DEG were considered significant if p-value was ≤ 0.01 and fold change of >1.5 or <-1.5.
Regulatory network prediction was accomplished by use of GeneMANIA plug-in for Cytoscape. Based on this database, an analysis of genes interacting with adherens junction protein coding genes was carried out.

### GO term enrichment

Genes were analysed for GO biological process enrichment, using the online annotation database: DAVID Bioinformatics Resources 6.8 (Huang et al., 2008). Functional annotation clustering with default settings was used; medium stringency and Benjamini–Hochberg correction was applied. The enriched GO terms with FDR <0.05 were selected and displayed in the interaction map.

### Calculations and statistical analysis

Excel and XLstat were used to perform calculations, generate graphs and calculate statistical significance with Student's t-test, Mann-Whitney U test, Kruskall-Wallis test, and Spearman Correlation test where P>0.05 was considered not significant, *P<0.05, **P<0.01, ***P<0.001, ****P<0.0001 and $r_s \in [0.5, 1.0]$ was considered to suggest a strong, $r_s \in [0.3, 0.5]$ a moderate, $r_s \in [0.1, 0.3]$ a weak and $r_s \in [0, 0.1]$ a very weak or non-existent correlation.

Supplemental References

HUANG, D. W., SHERMAN, B. T. & LEMPICKI, R. A. 2008. Systematic and integrative analysis of large gene lists using DAVID bioinformatics resources. *Nature Protocols,* 4, 44.
